# Plant domestication shapes rhizosphere microbiome assembly and metabolic functions

**DOI:** 10.1186/s40168-023-01513-1

**Published:** 2023-03-31

**Authors:** Hong Yue, Wenjie Yue, Shuo Jiao, Hyun Kim, Yong-Hwan Lee, Gehong Wei, Weining Song, Duntao Shu

**Affiliations:** 1 State Key Laboratory of Crop Stress Biology in Arid Areas, College of Agronomy, Northwest A&F University, Yangling, Xianyang, 712100 Shaanxi China; 2 Shaanxi Key Laboratory of Agricultural and Environmental Microbiology, Yangling, Xianyang, 712100 Shaanxi China; 3 State Key Laboratory of Crop Stress Biology in Arid Areas, College of Life Sciences, Northwest A&F University, Yangling, Xianyang, 712100 Shaanxi China; 4 Department of Agricultural Biotechnology, Seoul National University, Seoul, 08826 Korea

**Keywords:** Plant domestication, Root exudation, Rhizosphere microbiomes, Microbial interaction network, Microbial metabolic functions, Wheat

## Abstract

**Background:**

The rhizosphere microbiome, which is shaped by host genotypes, root exudates, and plant domestication, is crucial for sustaining agricultural plant growth. Despite its importance, how plant domestication builds up specific rhizosphere microbiomes and metabolic functions, as well as the importance of these affected rhizobiomes and relevant root exudates in maintaining plant growth, is not well understood. Here, we firstly investigated the rhizosphere bacterial and fungal communities of domestication and wild accessions of tetraploid wheat using amplicon sequencing (16S and ITS) after 9 years of domestication process at the main production sites in China. We then explored the ecological roles of root exudation in shaping rhizosphere microbiome functions by integrating metagenomics and metabolic genomics approaches. Furthermore, we established evident linkages between root morphology traits and keystone taxa based on microbial culture and plant inoculation experiments.

**Results:**

Our results suggested that plant rhizosphere microbiomes were co-shaped by both host genotypes and domestication status. The wheat genomes contributed more variation in the microbial diversity and composition of rhizosphere bacterial communities than fungal communities, whereas plant domestication status exerted much stronger influences on the fungal communities. In terms of microbial interkingdom association networks, domestication destabilized microbial network and depleted the abundance of keystone fungal taxa. Moreover, we found that domestication shifted the rhizosphere microbiome from slow growing and fungi dominated to fast growing and bacteria dominated, thereby resulting in a shift from fungi-dominated membership with enrichment of carbon fixation genes to bacteria-dominated membership with enrichment of carbon degradation genes. Metagenomics analyses further indicated that wild cultivars of wheat possess higher microbial function diversity than domesticated cultivars. Notably, we found that wild cultivar is able to harness rhizosphere microorganism carrying N transformation (i.e., nitrification, denitrification) and P mineralization pathway, whereas rhizobiomes carrying inorganic N fixation, organic N ammonification, and inorganic P solubilization genes are recruited by the releasing of root exudates from domesticated wheat. More importantly, our metabolite-wide association study indicated that the contrasting functional roles of root exudates and the harnessed keystone microbial taxa with different nutrient acquisition strategies jointly determined the aboveground plant phenotypes. Furthermore, we observed that although domesticated and wild wheats recruited distinct microbial taxa and relevant functions, domestication-induced recruitment of keystone taxa led to a consistent growth regulation of root regardless of wheat domestication status.

**Conclusions:**

Our results indicate that plant domestication profoundly influences rhizosphere microbiome assembly and metabolic functions and provide evidence that host plants are able to harness a differentiated ecological role of root-associated keystone microbiomes through the release of root exudates to sustain belowground multi-nutrient cycles and plant growth. These findings provide valuable insights into the mechanisms underlying plant-microbiome interactions and how to harness the rhizosphere microbiome for crop improvement in sustainable agriculture.

Video Abstract

**Supplementary Information:**

The online version contains supplementary material available at 10.1186/s40168-023-01513-1.

## Background

Plant domestication, a complex evolutionary process, is a crucial accomplishment in human history [57]. It provides a continuous food supply and enhances the establishment of stable human settlements and has far-reaching consequences for human prosperity. The first crop plants were subjected to domestication ~ 13,000 years ago, and a plethora of crop plants, such as rice, wheat, and barley, have been undergoing continuous domestication by anthropogenic intervention in modern agricultural areas [50]. The improvement of phenotypic traits of domesticated cultivars not only is determined by their own genetic features [53] but also depends on their root-associated microbial communities. Pioneering studies have indicated that plant domestication profoundly influences seed and root microbiome assembly [37, 39], particularly the rhizosphere microbiome [69]. These affected rhizosphere microbiomes have important roles in crop performance and growth [74, 84]. Hence, the dissection of mechanisms underlying the species coexistence of these affected microbiomes and plant-microbiome interactions at the root-soil interface will provide new avenues for harnessing indigenous rhizobiomes to promote soil health and crop production [10, 66].

In addition to being subjected to the selective power of domestication, root-associated microbiomes are largely affected by various root exudates that are released by distinctive genotypes of plants [55]. Root exudates include amino acids, sugars, organic acids, nucleotides, fatty acids, hormones, and secondary metabolites, which have direct and indirect impacts on nutrient availability in the rhizosphere [3]. Increasing evidence highlights that host plants are able to harness root-associated microbiomes by releasing root exudates for plant nutrient acquisition [38, 41, 49] and mitigating abiotic [11] and biotic stresses [32] during plant domestication processes [63]. These studies highlight the importance of root exudation in shaping root-associated microbial communities under field and experiment conditions [34, 63]. However, we still lack comprehensive knowledge of how and to what degree crops under distinct domestication statuses control root exudation to shape rhizosphere microbiomes with specialized metabolic functions.

The aforementioned plant-microbiome associations along the soil-root continuum not only are affected by root exudation but also are also profoundly influenced by microbe-microbe interactions. Emerging studies have indicated that these interactions are now recognized to implement crucial functions for promoting plant biodiversity [75] and plant survival [17] and protect hosts against soilborne pathogen infection [83]. Importantly, the putative keystone taxa that strongly interact with other species in the microbial interkingdom network are suggested to play crucial roles in enhancing microbial network complexity [4, 77], crop production [20, 73], and ecosystem multi-functionality [35]. However, knowledge is lacking on how these complex microbial interkingdom interactions and putative keystone taxa respond to plant domestication.

Domesticated emmer wheat (*Triticum turgidum ssp. dicoccon*) evolved from wild emmer wheat (*Triticum turgidum ssp. dicoccoides*), which was subjected to domestication that began ~ 10,300 years ago in the Fertile Crescent [45]. The domestication of emmer wheat was essential to human prosperity but also led to significant changes in wheat genetic diversity [47]. Understanding the genetic consequences of the evolution and domestication history of tetraploid wheat in root-associated microbial community assembly and functions will pave the way for precise plant breeding strategies and soil health. Nevertheless, studies assessing the mechanisms of species coexistence and functional adaptation in response to the domestication of tetraploid wheat remain scarce. To fill this knowledge gap, the approach of “going back to the roots” was adopted, and 44 accessions of tetraploid wheat, including wild emmer and domesticated emmer, were selected in the current study. Herein, our aims were to (1) uncover the adaptive strategies and interkingdom interaction patterns of the root-associated microbiome during the wheat domestication process, (2) decipher the functional traits of the rhizosphere microbiome related to nutrient acquisition and dissect how the root exudation covaries with aboveground plant phenotypes in response to plant domestication, and (3) disentangle the crucial role of rhizosphere microbial consortium and individual keystone taxa in sustaining root morphology traits.

## Materials and methods

### Selection of plant accessions

Grains of 44 accessions of tetraploid wheat, i.e., 22 wild and 22 domesticated wheat, were obtained from the International Wheat Genome Sequencing Consortium (IWGSC) on the basis of our previous study [13]. Seeds of the 44 tetraploid wheat accessions were planted annually for field experiments since 2014 at Caoxingzhuang Agro-Ecosystem Experimental Station of the Northwest Agriculture and Forest University, Shaanxi Province, China (34°17′N, 108°04′E). In the last 9 years, new seeds of the 44 tetraploid wheat accessions from the current year were planted annually for the current planting season. For winter wheat and fallow system, the summer fallow period of winter wheat is from late July to late September. The seeds of the 44 tetraploid wheat accessions were annually collected at the maturity stage (June 15th-June 30th). Then, these collected new seeds are continued to sow in the middle of October of current year. After nine times of planting season, the 44 tetraploid wheats were chosen for further use. At the heading stage of wheat, five replicates of each wheat accession were randomly chosen to measure the plant attributes, including plant height, ear length, subsegment length, and tiller (Table S1).

**Fig. 1:**
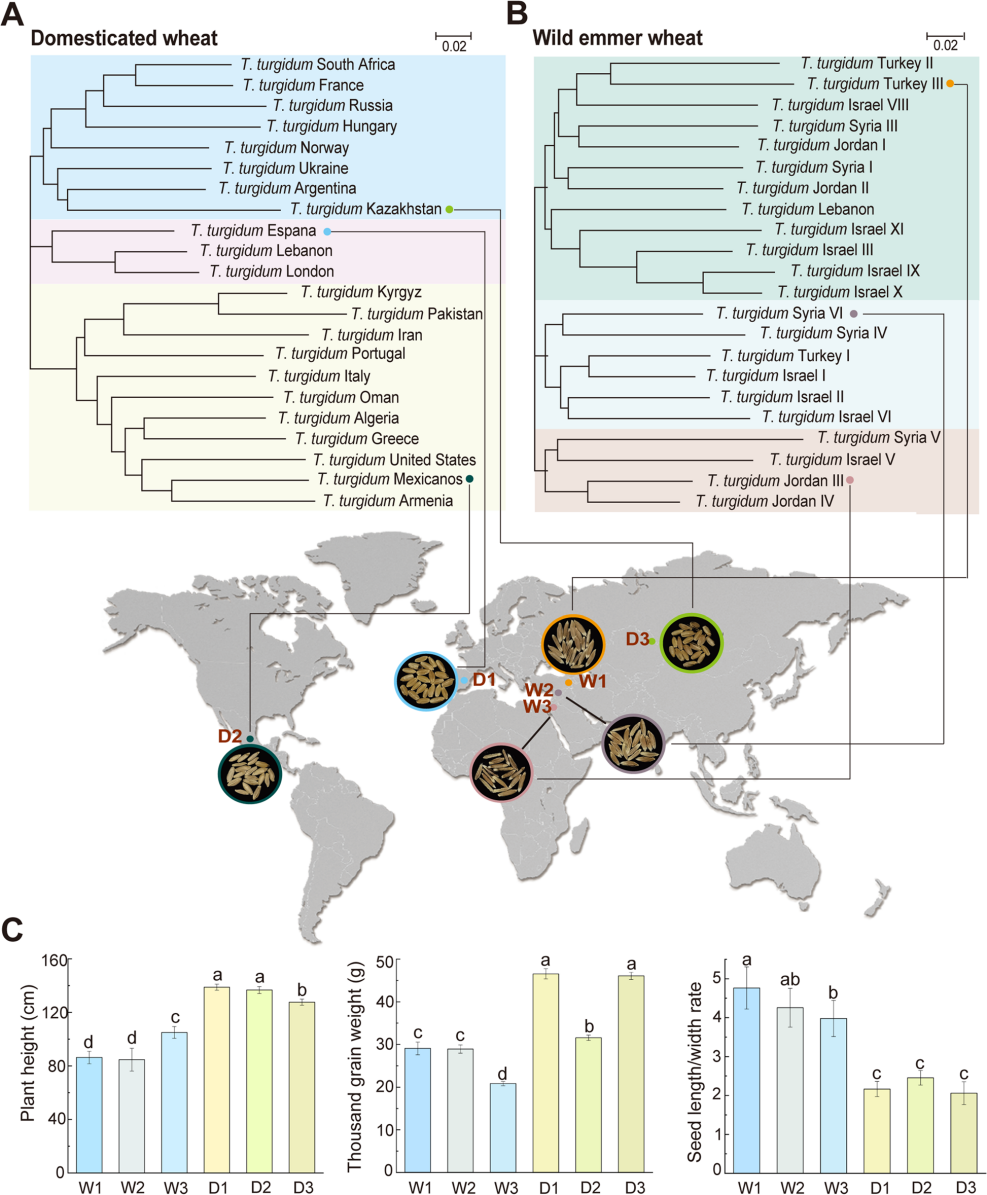
The worldwide distribution and plant phenotypes of domesticated and wild tetraploid wheats. **A**, **B** Phylogenetic tree of wheat samples and geographic distribution. The phylogenetic tree of tetraploid wheat genotypes was constructed based on their whole-genome sequences that were obtained from International Wheat Genome Sequencing Consortium (IWGSC). MEGA-X program was applied to generate neighbor-joining tree with 1000 bootstraps. **C** Three domesticated accessions (*T. turgidum* ssp. *dicoccon*) including D1 from different wheat-growing regions of España, D2 (Mexicanos), and D3 (Kazakhstan) and three wild emmer accessions (*T. turgidum* ssp. *dicoccoides*) including W1 (Turkey), W2 (Syria), and W3 (Jordan) were calculated plant phenotypes like plant height, thousand grain weight, and ratios of seed length and width at the heating stage of these wheats. Error bars represent standard errors (*n *= 4). Different lowercase letters above the bars indicate significant differences (*P* < 0.05), based on Kruskal–Wallis test

Based on the phylogenetic tree of 44 tetraploid wheats that were captured from whole-genome sequences (Fig. 1), the individual accessions with representative branches in the phylogenetic tree and the best plant performance of phenotypes, including three domesticated accessions D1, D2, and D3 from different wheat-growing regions of Kazakhstan, Espana, and Mexicanos and three wild emmer accessions W1, W2, and W3 from different wheat-growing regions of Turkey, Syria, and Jordan in six distinct branches, were further selected for this study (Table S2). More details for experiment design and field site management can be found here (Supplementary Notes: Method S1).

### Samples collection, DNA extraction, PCR amplification, and amplicon sequencing

At the maturity stage on June 15, 2021, 60 soil samples were collected (5 replicates × 6 plant accessions × 2 compartments; Supplementary Notes: Method S2). All soil samples were stored at − 30 °C until further use. Additionally, the harvested seeds of each wheat accession were further collected to measure their phenotypic attributes. The total DNA from collected soil samples was separately extracted using the PowerSoil DNA Isolation Kit (Mo Bio Laboratories Inc., Carlsbad, CA, USA), according to the manufacturer’s instructions. For PCR amplification, the universal 515F and 806R PCR primers with barcodes were applied to amplify the V4 regions of 16S rRNA. The PCR primers ITS1F and ITS2R were used to amplify the ITS1 regions of fungal 18S rRNA. The PCR protocols for constructing 16S rRNA and fungal ITS libraries followed the manufacture’s instructions. After amplification, PCR products were pooled and purified with an AxyPrep DNA Gel Extraction Kit (Axgen, USA). Finally, the generated amplicon libraries were sequenced on an Illumina HiSeq 2500 PE250 platform (Majorbio, Co., Ltd., China). Following sequencing, the demultiplexing sequences, merging reads, and removing non-biological sequences for all raw reads were performed using the QIIME2 (2021.8, https://QIIME2.org)-CentOS 7.6 platform (Supplementary Notes: Method S2).

### Shotgun metagenomics sequencing

The extracted DNA for amplicon sequencing were also used for metagenomics sequencing on an Illumina HiSeq 2500 Platform (150 bp paired-end reads). The raw metagenomics sequences were quality filtered using fastp (v0.23.1) based on a minimum Q Score of 20 and a minimum sequence length of 50 bp [12]. The obtained clean reads were assembled into scaffolds individually using IDBA-UD v1.1.1 with default parameters [52]. Scaffolds longer than 500 bp were used to predict open reading frames (ORFs) using MetaGeneMark (v0.43) [85]. The generated ORFs were then clustered at 95% similarity to construct a nonredundant gene catalog using MMseqs2 [67]. The nonredundant gene catalog was also searched against the eggNOG database (v5.0) [33] and Kyoto Encyclopedia of Genes and Genomes (KEGG) database (release 80.1) [36] using DIAMOND (v0.922.123), with an *E*-value < 1e−10 to annotate the predicted genes to clusters of orthologous groups (COG) and KEGG Orthology (KO) groups. For taxonomic assignment (Supplementary Notes: Method S3), the nonredundant genes were searched against the NCBI NR database (release March 15, 2020) using DIAMOND (v0.922.123) with an *E*-value cutoff of 1e−5 [7].

### Root exudate collection and LC–MS analysis

After sampling of the rhizospheric soil, the intact root systems were used to collect root exudates according to a pioneering study with minor revision [16]. We totally collected 36 root exudate samples (6 replicates × 6 plant accessions). Prior to detection, root exudate powders were redissolved in 100-μL sterilized deionized water for downstream analysis (Supplementary Notes: Method S4). After detection, the metabolites in each sample with a relative standard deviation (RSD) > 30% were removed from the dataset. The mass spectra of remaining metabolites were annotated with the Golm Metabolome Database (GMD, http://gmd.mpimp-golm.mpg.de/), METLIN database (http://metlin.scripps.edu), and KEGG) database (release 80.1). Finally, the annotated metabolites were log-transformed for further microbiome-wide association study.

### Rhizospheric microbiota inoculation and plant morphology traits analysis

To verify whether rhizospheric microbiota change the root growth, the inoculation treatment with addition of live microbiota suspensions was conducted (Supplementary Notes: Method S5). The control treatment was denoted without addition of rhizospheric microbiota. Secondly, *Microbacterium mitrae* was selected to a follow-up experiment. Bacterial culture was normalized to OD600 = 0.2 for plant inoculation experiments. Control treatments received Hoagland nutrient solution instead of a bacteria suspension. Wheat seeds were surface sterilized with 75% ethanol for 30 s and 2.5% sodium hypochlorite for 15 min and then germinated on 0.5 × MS agar media for 5 days. Then, a 5-day-old sterile wheat seedlings were transferred to the Hoagland nutrient solution with or without inoculation microbial in the Magenta boxes at 27 °C with 16-h light/8-h dark cycle. The mixed nutrient solutions were changed at intervals of 3 days. Root-related parameters including root length, and number of root forks, etc., were scanned and registered using Microtek ScanMaker i800 plus system (WSeen, Hangzhou, China). The shoot fresh weights and dry weight of each seedling were measured after 15 days.

### Statistical analyses

The α-diversity and *β*-diversity were determined using the “vegan” R package [51], unless otherwise indicated. For *β*-diversity, the ASV table was normalized by the trimmed mean of *M*-values (TMM) method using the “EdgeR” bioconductor package [60]. The generated Bray–Curtis dissimilarity matrix retrieved from the “vegdist” function was used to perform further analyses, including an analysis of similarity (ANOSIM), principal coordinate analysis (PCoA), and permutational multivariate analysis of variance (PERMANOVA). To further confirm the constrained variables for the bacterial and fungal communities, constrained analysis of principal coordinates (CAP) was performed using the “capscale” function in the “vegan” R package [15] and the “ordinate” function in the “phyloseq” R package [48]. To determine the enriched and depleted ASVs in wild and domesticated wheat plants, differential abundance analysis was conducted using the generalized linear model (GLM) approach in the “EdgeR” package. More details for following analyses can be found in the here (Supplementary Notes: Method S6).

### Random forest classification and tenfold cross validation

To explore the importance of different microbial taxonomy categories in structuring wild/domesticated microbiome assembly, the “random forest (RF)” method was used to perform machine learning classification. This step was implemented using the “importance” function in the “randomForest” R package [59]. Subsequently, tenfold validation was performed to evaluate the accuracy of the RF model and to select the minimum number of ASVs with the lowest prediction error rate by using the “rfcv” function in the “randomForest” package. Finally, the 30 most important ASVs for bacteria and fungi from the RF model were categorized as wild/domesticated enriched and bulk/rhizosphere-enriched ASVs at the genus level depending on the results of the aforementioned differential abundance analysis.

### Microbial co-occurrence network analysis

To explore the microbial hierarchical interactions between bacterial and fungal communities in the rhizosphere of wild and domesticated wheats, we implemented correlation-based network analysis by using the FastSpar algorithm [78]. To minimize the bias of pairwise correlations, the core community for bacteria was generated based on the criteria of mean relative abundance and occurrence frequency of microbial communities in all samples. After that, core bacterial and fungal ASVs were merged to construct multi-kingdom ASV tables for further analysis. Only compositionality-robust (|*ρ*|> 0.7) and statistically significant (*q* < 0.01) correlations were integrated into the hierarchical network analysis. The node-level topological features including the degree, betweenness, closeness, and eigenvectors were calculated using the “igraph” R package [15]. The identification of keystone taxa was based on the criteria of the nodes with high degree, higher node transitivity, and low betweenness centralities. Finally, the generated co-occurrence network with keystone taxa and different modules was visualized using the Gephi platform (v0.92, https://gephi.org).

### Plant-microbiota-metabolite association analysis

We then implemented partial least squares discriminant analysis (PLS-DA) and orthogonal partial least squares discriminant analysis (OPLS-DA) by using the “opls” function in the “ropls” bioconductor package [71] to uncover the mechanisms that underlie the microbial response to plant domestication. Subsequently, ecological associations among plant features, significant metabolites, and enriched microbiota were calculated using the “rcorr” function in the “Hmisc” R package [28]. Correlation matrix visualization was performed using the “ggtree” [82] R package. Based on this association analysis, key relevant metabolites that were significantly enriched in wild or domesticated wheat were further used to evaluate their importance for plant growth. The importance of each metabolite was determined by evaluating the increase in the mean square error (MSE) between predictions and observations. This analysis was performed using the “rfPermute” and “rp. importance” functions in the “rfPermute” R package [2]. Meanwhile, the significance of the importance of each metabolite associated with plant traits was assessed by using the “rfUtilities” R package [19] The significance of this model was cross-validated using the “A3” R package [24].

## Results

### Distinct diversity patterns of soil microbial communities from wild and domesticated wheat

For all wheat genotypes, the bacterial community from domesticated wheat presented higher Shannon diversity than that from wild wheat, while the fungal community displayed the opposite pattern (Fig. S1 and Table S3). The multivariate linear regression analysis suggested that plant domestication status had a greater selection influence on both bacterial (*F* = 8.388, *P* = 0.005) and fungal Shannon–Wiener diversities (*F* = 7.501, *P* = 0.008) among different genotypes in the rhizosphere soils. For bulk soils, the analysis revealed that plant domestication status was also the main factor influencing α-diversity of both bacterial (*P* = 0.0029) and fungal (*P* = 0.0013) communities. Apart from affected by plant domestication and niche compartment, the genotype of host speciation could have potentially broader influences on bacterial (*R*2 = 17.2%, *P* < 0.001) and fungal communities (*R*2 = 12.8%, *P* < 0.001; Table S3). Considering *β*-diversity, bacterial and fungal communities displayed a marked dissimilarities in wild wheat compared with domesticated wheat on the basis of Bray–Curtis matrix (Fig. S2). Analogously, this distinct separation was also found in different niche compartments, such as bulk and rhizosphere soils (Table S4). Notably, CAP results further revealed a marked separation between the soil microbial communities of wild and domesticated wheat for both bacteria and fungi (*P* < 0.001; Figs. S3 and S4). Based on these results, domestication exerted stronger selection influences on the rhizosphere bacterial and fungal community assembly, although other processes, such as niche compartment and plant genotypes, were also significant for the microbial community variations.

### Domestication altered microbial taxonomic patterns and interkingdom co-occurrences

**Fig. 2:**
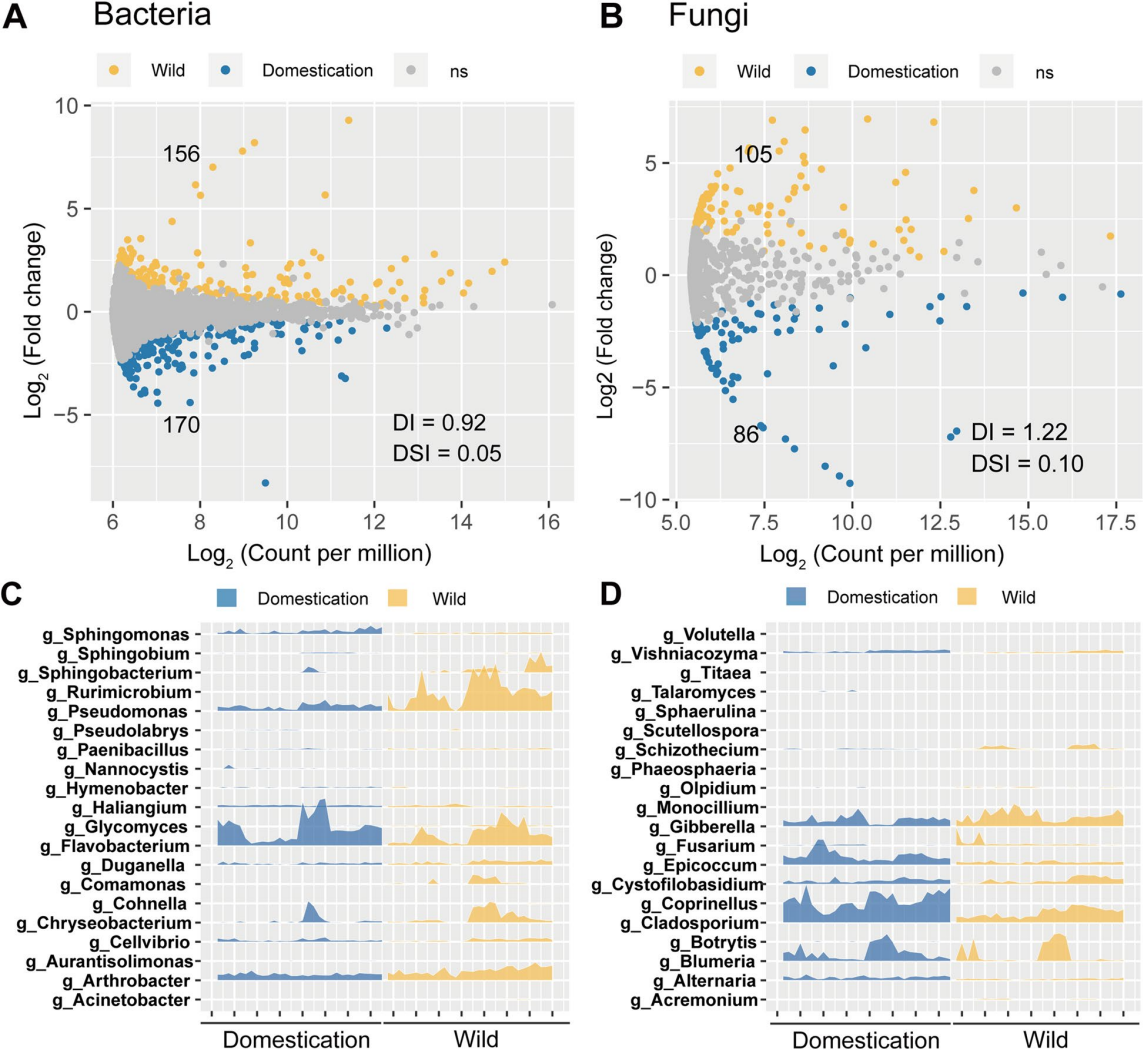
Rhizosphere amplicon sequence variants (ASVs) responsible for the community differences in the wild wheats and domesticated wheats that are calculated by a differential abundance test and random forest classification. **A** and **B** The volcano plot illustrating the enrichment and depletion patterns of rhizosphere bacterial and fungal microbiomes in the three wild wheats compared with three domesticated wheat accessions. The ASVs were colored by their categorization as “wild enriched,” “domesticated enriched,” and “non-differential” according to their values of Log2 (count per million) and Log2 (fold change). DI, depleted index; DSI, dissimilarity index. **C** and **D** Joyplots showing the relative abundance profiles of top 20 ASVs in bacterial and fungal communities that are revealed by a random forest (RF) classifier. The tenfold validation was performed to evaluate the accuracy of RF model and to select minimum number of ASVs with the lowest prediction error rate. The top ASVs on the genus level are listed along the y-axis representing their importance in contributing to the accuracy of domesticated and wild wheats prediction by calculating their mean decrease accuracy in the RF model

Taxonomic classification showed that both bacterial and fungal communities in the rhizosphere and bulk soils varied distinctly across wild and domesticated wheats (Fig. S5). Specifically, the differential abundance analysis indicated that 326 bacterial and 191 fungal ASVs from the rhizosphere were significantly affected by plant domestication status (Fig. 2A and B). Random forest modeling analysis further revealed that the top 30 enriched bacterial ASVs, mainly from the phyla Proteobacteria, Bacteroidetes, and Actinobacteria, were the most important predictors of plant domestication status from the rhizosphere (Fig. 2C). For the fungal community, the top 30 discriminant ASVs consisted of Ascomycota and Basidiomycota (Fig. 2D). The opposite enrichment patterns in the bulk soils were also observed between wild and domesticated wheat (Fig. S6). The marked differentially distributed ASVs showed asymmetric enrichment patterns in accordance with the plant domestication status and niche differentiation.

**Fig. 3:**
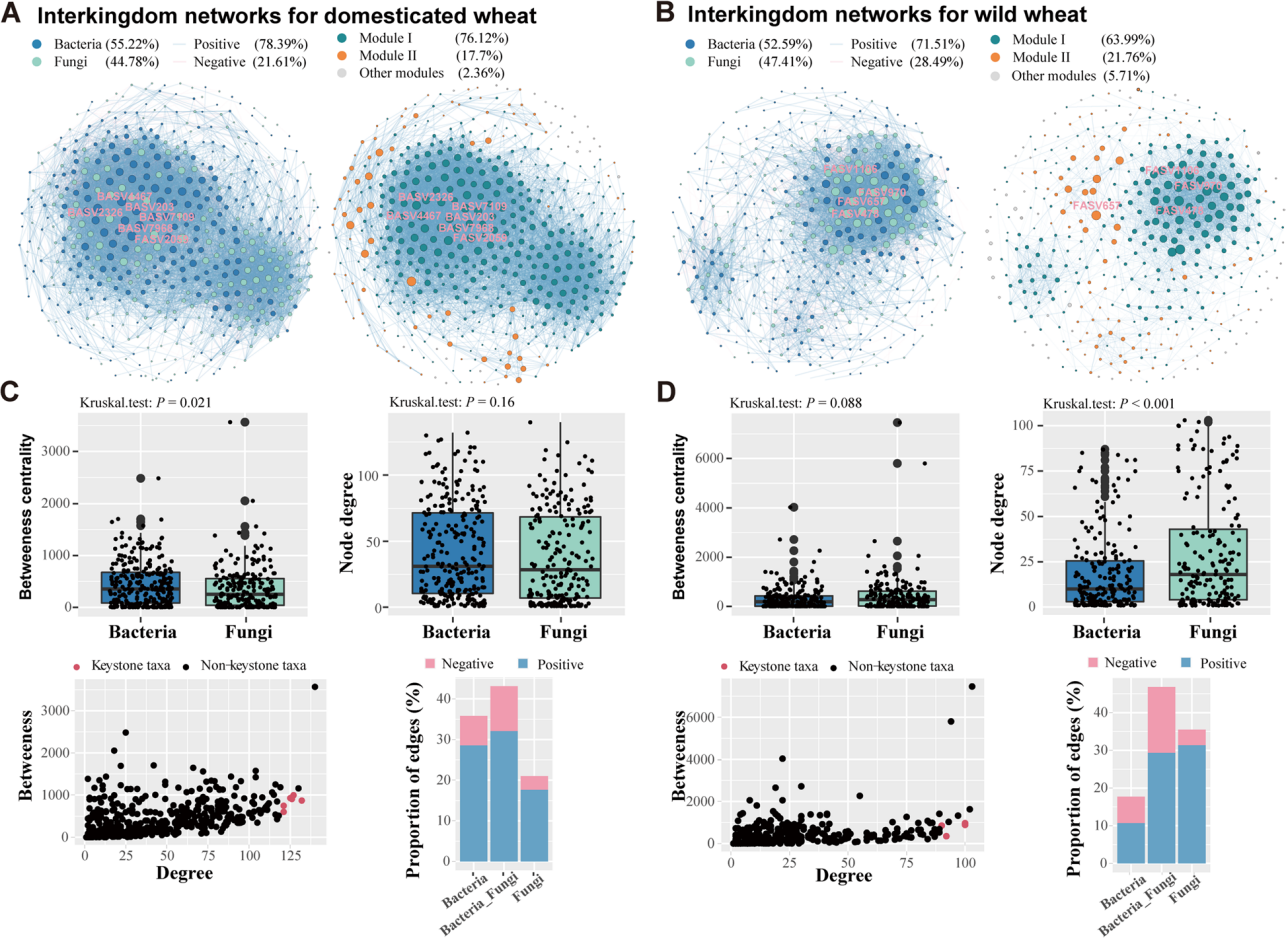
Rhizosphere microbial interkingdom association networks and node-level topological features. **A** and **B** Interkingdom co-occurrence networks in the domesticated and wild wheats. Only compositionality-robust (|*p*|> 0.7) and statistically significant (*q*<0.01) correlations were shown. The size of each node indicates the relative abundance of each ASV. The color of each node represents the bacteria or fungi taxa. Blue solid lines represent co-presence associations, and red line represents mutual exclusive correlations. The thickness of each link line is proportional to the correlation coefficients of the connections. The keystone taxa and dominant modules for each networks were also shown. **C** and **D** Box graphs illustrating the node-level topological features of each networks, including betweenness and degree. Comparison of these two features demonstrating the high degree and low betweenness for the keystone taxa. Bar diagrams showing the proportion of inter- and intra-kingdom edges of positive or negative correlations in the rhizosphere network. The significance of differences between domesticated and wild wheats was determined by Kruskal–Wallis test

The microbial co-occurrence network analysis revealed that bacterial-fungal interkingdom interaction patterns evolved clearly across wheat domestication, with discrepant bacterial and fungal roles in the wild and domesticated microbial network. Specifically, bacterial taxa from rhizospheric soils showed higher network connectivity than fungal taxa in the domesticated microbial network, while the pattern diverged greatly in the wild microbial network (Fig. 3A–D and Table S5). In addition, the network connectedness of bacteria to bacteria and bacteria to fungi were higher in the rhizospheric microbial network in domesticated wheat than in wild wheat, but network connectedness of fungi to fungi were higher in the wild network than in the domesticated network. Compared to the rhizosphere network, the opposite patterns were observed in the microbial network of bulk soils (Fig. S7 and Table S5). Notably, within the microbial interkingdom association network in the bulk soils, we found that plant domestication reduced the network connectivity, accompanied by lower proportion of associations of bacteria-fungi and fungi-fungi communities. Dominant taxa in different network modules confirmed the discrepant influences of wheat domestication on the multitrophic network (Fig. S8). These results indicated that wheat domestication enhanced the bacteria-bacteria intrakingdom and bacteria-fungi interkingdom associations but decreased the fungi-fungi associations in the rhizosphere.

We further defined potential “keystone taxa” based on the criteria of nodes with high values of degree, transitivity, and low betweenness centrality in the microbial interkingdom networks. It was evident that the keystone taxa of the rhizosphere microbial network in domesticated wheat were bacteria, whereas associations within the wild wheat microbial network were mostly clustered around the fungal network. (Fig. 3A and B). This observed contrast pattern suggests the distinct roles of fungi and bacteria in sustaining the microbial network of domesticated and wild wheat. Collectively, these results indicated that assemblage patterns and ecological associations of the microbial community in the rhizosphere of domesticated wheat were regulated by bacteria, whereas fungi were more important in the wild wheat.

### Domestication status affects the functional profiles of the rhizosphere microbiome

**Fig. 4:**
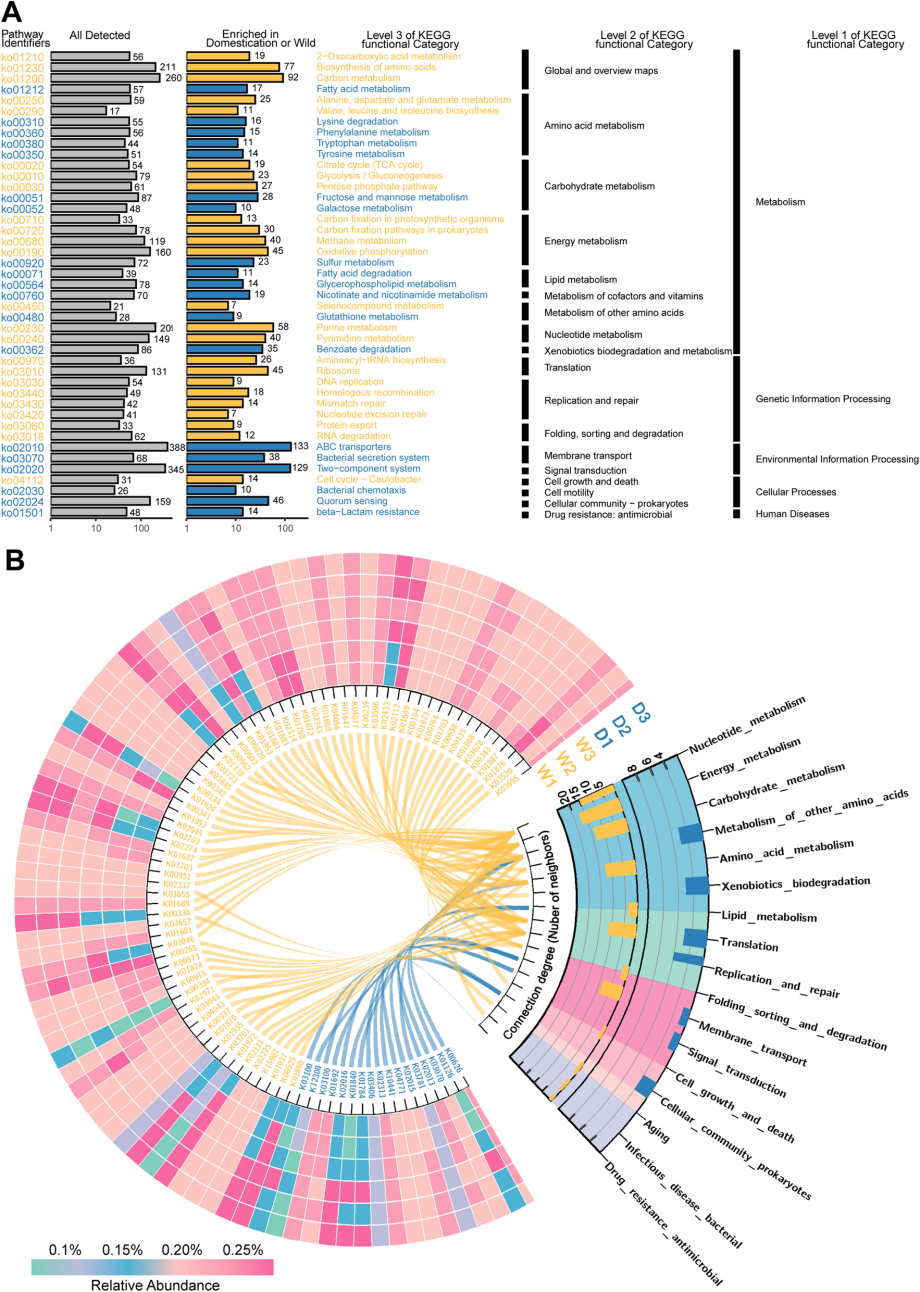
Functional profiles of rhizosphere microbiomes in the domesticated and wild wheats. **A** KO functional categories and pathways that were significantly enriched in domesticated wheats are marked in blue; those significantly enriched in wild wheats are marked in orange. All functional categories were determined using the two-tailed Wilcoxon test. **B** Distribution of enriched functional genes associated with different pathways. KO functional categories that were significantly enriched in domesticated or wild wheats were separately analyzed. Only the dominant KO functional categories (relative abundance > 0.07%) are shown in the histogram and are linked. The data were visualized using Circos (Version 0.69, http://circos.ca/)

Based on the metagenomics sequencing for rhizosphere samples from domesticated and wild wheats (Table S6), differential abundance analysis revealed that the number of significantly enriched KO functional categories in wild wheat was higher than that in domesticated wheat (Wilcox test, *P* < 0.05, Fig. S9). Notably, we found that “translation,” “replication and repair,” and “folding, sorting, and degradation” functional categories affiliated to “genetic information processing” pathway were only enriched in the wild wheat (Fig. 4A and Table S7). In the differential KO functional categories enriched in domesticated wheat, the functional gene (K03406) affiliated with methyl-accepting chemotaxis protein (MCP) was the most abundant KO functional category, followed by K00626 (atoB, acetyl-CoA C-acetyltransferase) and K03070 (secA, preprotein translocase subunit). These functional categories are involved in “two-component system,” “fatty acid degradation,” and “quorum-sensing” pathways (Fig. 4B). In contrast, KO functional categories involved in “nucleotide excision repair” and “purine metabolism” pathways were labelled as dominant genes in wild wheat, such as K03657 (pcrA, ATP-dependent DNA helicase), K03701 (urvA, excinuclease ABC subunit A), and K03046 (rpoC, DNA-directed RNA polymerase subunit beta).

NMDS ordinations of the KEGG Orthology, CAZyome, and COG profiles indicated that the functional position of rhizosphere microbiome of the domesticated wheat significantly differed from that of wild wheat (*P* < 0.001), and domestication status also had a significant influence (*R*2 = 26.2%, *P* = 0.015) on rhizosphere microbiome functions (Fig. S10A). Notably, the domesticated wheat possessed higher microbiome functional diversity than that of wild wheat. Moreover, a whole bunch of functional genes associated with C, N, and P cycling were dominantly enriched in wild wheat. In terms of C cycling (Fig. S10B), functional genes involved in carbon fixation (e.g., *pccA* and *smtA*) were more abundant in wild wheat. Regarding N cycling (Fig. S11A), nitrate reduction genes (e.g., *narG* and *narH*) and nitrogen assimilation gene (*nasA* and *nasB*), and denitrification genes (e.g., *nirS* and *nosZ*), were more abundant in wild wheat, while nitrogen fixation genes (e.g., *nifH* and *nifK*) and organic nitrogen metabolism gene (*gdhA*) were more abundant in domesticated wheat. For P cycling (Fig. S11B), wild wheat enriched more functional genes related to organic P mineralization (e.g., *phnX* and *ugpQ*) and P transport system (e.g., *phnC* and *phnE*), while functional genes affiliated with inorganic P solubilization (*gcd*) were more abundant in domesticated wheat. Additionally, we found that functional genes (i.e., K02314, K01687, and K04066) associated with C, N, and P cycles were important predictors of plant phenotype traits, including plant height, chlorophyll content, and ear length (Fig. S12).

### Root metabolite traits and their links with the microbial community and plant phenotype profiles

Metabolomics analysis revealed that root exudates exhibited significant differences between wheat with different domestication statuses, reflecting dissimilar metabolic profiles in wild and domesticated wheat (Fig. S13). More importantly, metabolites such as oleamide, apiin, and xanthine were more abundant in the wild wheat, while metabolites such as malic acid, leucodopachrome, and postin were enriched in the domesticated wheat (Fig. S14). These results indicated that divergences in metabolites were highly correlated with plant domestication and that this activity led to a decline in metabolite diversity in plant breeding history.

**Fig. 5:**
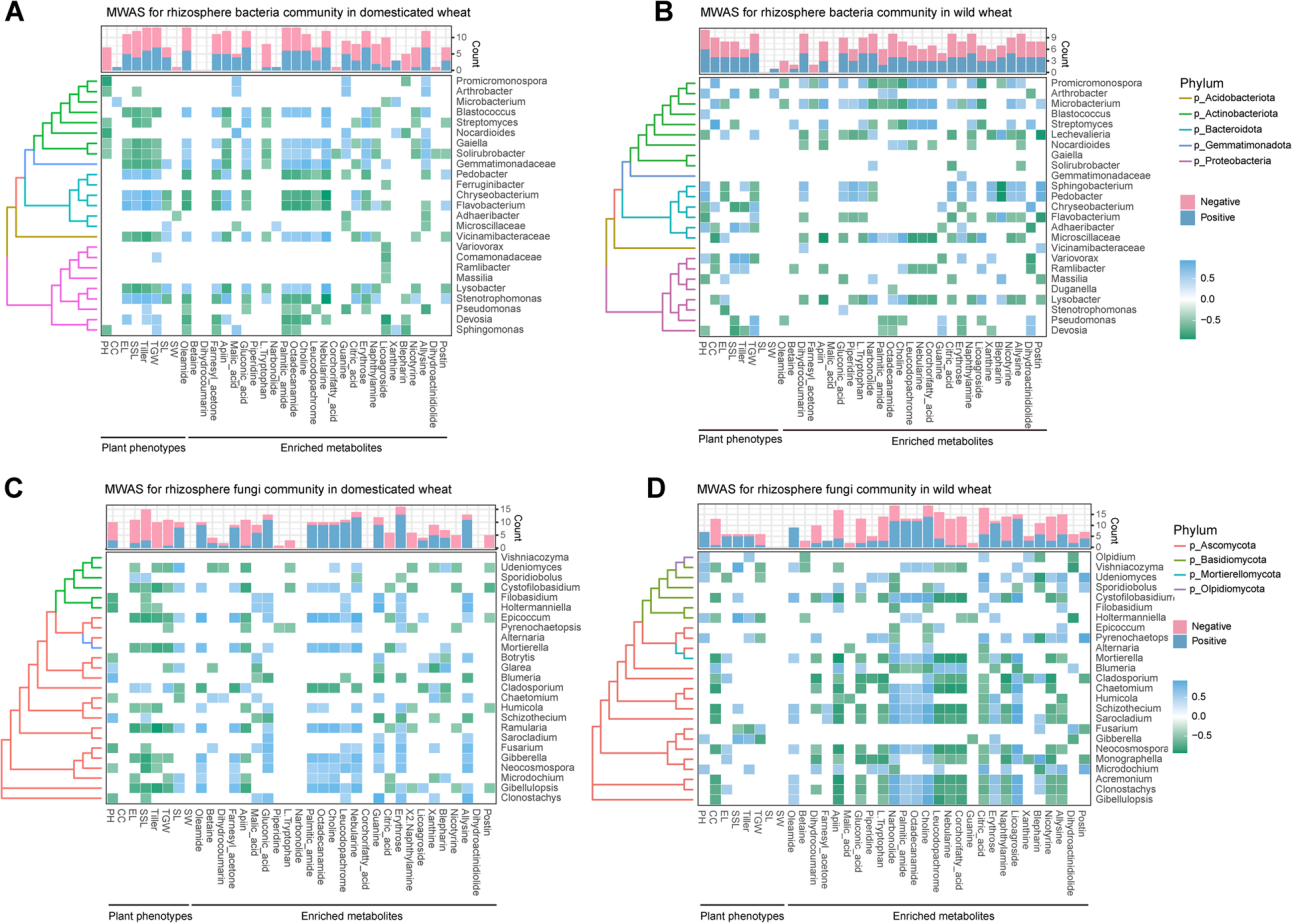
**A**–**D** Metabolite-wide association study (MWAS) for enriched rhizosphere bacteria and fungi in domesticated and wild wheats. Heatmap illustrating the statistically significant and positive associations among rhizosphere-enriched ASVs, aboveground plant phenotypes, and enriched metabolites, with associated phylogenetic tree of obtained ASVs from SILVA SSU 138. Only ASVs that were correlated with both phenotypes and root exudates with correlation coefficient |*ρ*|> 0.5. Each tip indicates a single ASV labelled according to the genus name. The color of branch in the phylogenetic tree represents phyla. Histogram on the top shows the number of positive (marked red) or negative (marked blue) associations registered for each variable. PH, plant height; CC, chlorophyll content; EL, ear length; SSL, subsegment length; tiller; TGW, thousand grain weight; SL, seed length, SW, seed width

We further used a metabolite-wide association study (MWAS) to investigate the specific response of dominant microbial taxa to biomarker metabolites as well as their links with plant phenotype. For domesticated wheat, the top 25 dominant bacterial genera and fungal genera had more negative associations and positive correlations with enriched metabolites, respectively (Fig. 5). However, these patterns were reserved in the wild wheat. In regard to the associations between metabolites and plant phenotype, random forest analysis revealed that these associations were robust and some metabolites involved in malic acid, corchorifatty acid, postin, apiin, and octadecanamide were important predictors of plant attributes (Fig. S15). Regarding the links between plant attributes and dominant taxa, plant height of domesticated wheat had more positive correlations with some dominant bacterial and fungal species (Fig. 5A and C), while the patterns were reserved in wild wheat (Fig. 5B and D). These results based on the MWAS analysis revealed that distinct domestication statuses corresponded to the divergent ecological associations between bacterial and fungal communities, indicating their contrasting substrate preferences.

**Fig. 6:**
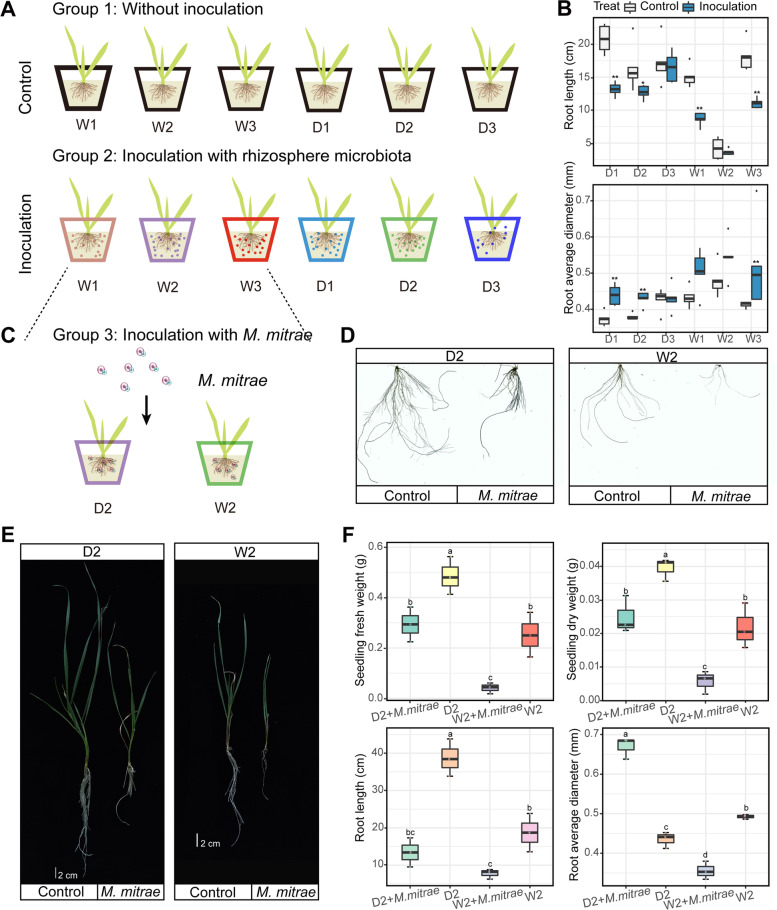
Response of root morphology traits after rhizosphere microbiota inoculation. **A** Microbial inoculation experiment for domesticated and wild wheat. Group 1, all selected wheat accessions without any addition of inoculum suspension. Group 2, all accessions were inoculated with corresponding inoculum suspension that was generated by mixing rhizospheric soil into TSB medium. Five replicates were set for all treatments. **B** Root length and root average diameter in the domesticated and wild wheat under the control and inoculation treatments. Error bars represent standard errors (*n* = 5). The significance of differences between group 1 and group 2 was determined by Kruskal–Wallis test. **C** Inoculation experiment of group 3 that only contains bacterial isolates (*Microbacterium mitrae*). **D** Root architecture traits under control and inoculation treatments were scanned and measured using Microtek ScanMaker i800 plus system. **E** Seedling fresh/dry weight and root morphology traits were measured. Error bars represent standard errors (*n* = 5). Different letters indicate significantly different groups (*P *< 0.05, Kruskal–Wallis test)

To further corroborate the links between plant attributes and microbial taxa based on the field data analysis, we performed a microbiota inoculation experiment to assess the impact of the bacterial culture or inoculum on plant growth (Fig. 6A; Table S8). The results showed that the inoculated rhizosphere microbiota significantly decreased root length but promoted root average diameter compared to the control treatment, regardless of plant domestication status (Fig. 6B). We further found that biodiversity of keystone phylotypes involved in bacterial genera *Nocardioides* (*R*2 = 0.529, *P* = 0.0012) and fungal genera *Holtermanniella* (*R*2 = 0.671, *P* = 1.1e-04) was significantly associated with the root length of domesticated wheat. For wild wheat, we also found that the biodiversity of *Pedobacter* (*R*2 = 0.656, *P* = 1.5e-04) and *Microdochium* (*R*2 = 0.746, *P* = 2.03e-05) was significantly correlated with root length (Fig. S16). Based on the results from rhizosphere microbiota inoculation experiment, we further verified *Microbacterium mitrae* significantly affected root growth of domesticated and wild wheat (Fig. 6C, D, and E). Regarding the root morphological traits, the inoculation of *M. mitrae* exerts inhibition root growth and reduction seedling fresh/dry weight (Fig. 6F). This inhibition effects can be eliminated with removing *M. mitrae*. Considering the ecological role of putative keystone taxa (e.g., *M. mitrae*) in maintaining the root morphological traits, these results suggest that individual keystone taxa led to a consistent growth regulation of root regardless of wheat domestication status.

## Discussion

### Plant domestication strongly influences the rhizosphere microbial communities and simultaneously decreases the functional diversity related to multiple nutrient cycles

Deciphering how plant domestication affects rhizosphere microbiome assembly and functions is of great importance to reinforce the plant-microbiome interactions under the framework of “going back to the roots” [54, 55]. Our results demonstrated that changes in the wheat rhizosphere microbiome were largely influenced by plant domestication status. Moreover, rhizosphere microbiomes were more sensitive to plant domestication than soil microbiomes. This finding is in line with previous studies conducted with *Arabidopsis* [64], barley [8], rice [72], and soybean [65], indicating that plant domestication is a crucial factor in shaping the assembly of the plant rhizosphere microbiome.

Metagenomic analyses in our study revealed that functional genes, which encode MCPs associated with “signal transduction” pathway, were enriched in the domesticated cultivars. MCPs have been found in plant-growth-promoting rhizobacteria such as the genera of *Pseudomonas* [26, 62], which were also markedly enriched in the rhizosphere of wheat in our study. The beneficial bacteria would use MCPs to detect specific concentrations of molecules in the extracellular matrix, enabling directional accumulation of the bacteria to the plant, and in turn protect the plant against agricultural activity stresses. The findings suggest that MCP gene enrichment in domesticated plants is likely associated with the accumulation of beneficial bacteria. These harbored bacteria can deploy plant-release signal molecules in the extracellular matrix, such as volatile organic compounds, oxalic acid, trehalose, glucose, or thiamine [29], triggering detected changes between rhizospheric bacteria and fungi and promoting interkingdom associations via host-to-microbe, microbe-to-microbe, and microbe-to-host interactions [25].

Furthermore, some functional genes involved in the C, N, and P cycles were the best predictors for the plant phenotype, suggesting that high gene diversity in the rhizosphere microbiome of wild wheat could ensure that host plants have a better phenotype with multiple nutrient cycles. The potential explanation is attributed to the highly functional diversity of microbial communities, which tend to possess greater functional redundancy and more complex interkingdom interactions [44, 76, 77]. In order to adapt the environmental perturbations and selection pressure of anthropological activities, domesticated plants exhibit a trade-off between high yields and the gene diversity of belowground microbial communities [54]. Thus, it would not be surprising to find that plant domestication decreased both microbial and functional diversity. Collectively, these findings suggest that plant domestication has significant implications for soil biogeochemical processes, including carbon fixation, nitrate reduction, and organic phosphate mineralization.

### Plant domestication shifts rhizosphere microbial communities from slow growing and fungi dominated to fast growing and bacteria dominated

Bacteria and fungi likely coevolved and interacted in soils prior to plants colonizing terrestrial ecosystem ~ 450 mya [29]. In this study, the increasing enrichment of the bacteria/fungi ratio suggests that rhizosphere bacteria became the dominant microorganisms in the domesticated wheat, while fungi played a more important ecological role in the wild wheat. This phenomenon is likely attributed to fungi favoring the habitat niches that maintained by the wild wheats and their associated root exudates, even though in the fertile conditions. Additionally, plant domestication was also accompanied by considerable habitat specialization and management practices, with gradually increased reliance on the inputs of agricultural activities (e.g., fertilizers) to acquire higher crop productivity and to mitigate abiotic and biotic stresses on the domesticated crops [54, 58]. The transition of plants from native habitats with low input activities to highly managed agricultural soil also results in substantial changes in microbial community and has further hampered beneficial interactions between plant and microbiome.

Network analysis provide comprehensive insights into the stability of microbial communities under environmental stresses, although this approach has some limitations [21]. In this study, domestication decreased the stability of microbial networks. The less stable interkingdom interactions in the rhizosphere microbial community of wheat are likely associated with long-lasting legacies from agricultural management practices [42], ecological competition at the phylogenetic level [31], and lower modularity [4, 27]. Consistent with a previous study [37, 61], the enriched keystone bacterial taxa in the present study mostly belonged to bacterial community, whereas keystone fungal taxa were associated with fungal community. This is likely due to the bacteria and fungi adopt distinct life history strategies to confront natural and anthropogenic stress during plant domestication [46].

Since microbial carbon fixation and carbon degradation processes are regulated by autotrophic and heterotrophic microorganisms, respectively, our results suggest that domestication shifts rhizosphere microbial communities from slow-growing autotrophic microorganisms to fast-growing decomposers or carbon utilizers. Additionally, we found that carbon fixation and degradation genes were both significantly associated with multiple edaphic variables (Table S9 and Fig. S17). Consistent with pioneer studies [14, 70], our findings reinforced that soil nutrients, particularly nitrogen, may greatly contribute to carbon flow at the soil-root interface. More importantly, our present study indirectly verified these shifts partly due to the release of distinct root exudates from domesticated and wild wheat. Pioneer studies highlighted how root exudates affect evolution of the crop rhizosphere [34, 56], indicating that plant hosts can modulate nutrient conditions and energy flow to control an ever-evolving microbial community in the rhizosphere via plants actively release exudates. The root exudates could attract and selectively enrich for specific metabolic functions of microbial species. Collectively, these findings suggest that domestication shifts rhizosphere microbial communities from fungi dominated and slow growing to bacteria dominated and fast growing, thereby resulting in a shift from fungi-dominated membership with enrichment of C fixation genes to bacteria-dominated membership with enrichment of C-degradation genes. This finding supported the notion that bacteria and fungi are relatively more important game-changers in carbon utilization and carbon sink, respectively [81].

### Contrasting functional roles of root exudates in shaping rhizosphere microbiome and determining root morphology traits

Deciphering the ecological role of root exudates will advance our understanding of belowground economic traits and provide valuable insights into the manipulation of the rhizosphere microbiome for sustainable agriculture [30, 74]. Our results in the present study indicated that plant domestication status determined the type of root exudate release. These root exudates exert selection pressures on rhizosphere microbiome that lead to differential effects on host performance and root morphological traits via host-to-microbe, microbe-to-microbe, and microbe-to-host feedback [25]. However, these positive or negative associations between root exudates and root morphology traits do not hold invariably [68, 80]. The underlying mechanisms by which contrasting roles of plant-derived metabolites shape the assembly of rhizosphere microbiome currently include the following: (i) modulation of root morphology (number and length of roots, root hairs, root diameter, and root surface), which directly determined whether the rhizosphere microbiome is successful in colonization on the root surface [79]; (ii) regulation of microbial intrakingdom or interkingdom interactions, which determined the complexity and stability of rhizosphere microbiome [17, 22, 63]; and (ii) formation of nutritional interdependencies, which determined the reciprocal exchange of metabolites between metabolic deficiency microbiome and molecular compounds from root exudates [29].

Given plant-soil feedback encompasses host-to-microbiome, microbiome-to-microbiome, and microbiome-to-host interactions [25], the host plants and rhizosphere microbiome are not an independent evolutionary unit but acting with a common interest, particularly for nutrient resource availability in agricultural intensive environment, which in turn modulates root trait variation via divergent belowground resource-acquisition strategies, such as the nutrient acquisition strategies, and includes nutrient-scavenging and nutrient-mining strategies [79]. Pioneering studies contend that root exudates are a colossal of the carbon pool [18]. From a cost–benefit perspective, the nutrient-scavenging pathways are an energy consumption process that requires significant carbon investment from increased root exudate release. The benefits are numerous, but plants may reallocate the release of root exudates to trade off against root growth and nutrient acquisition. Therefore, it would not be surprising to find that domesticated and wild wheat exhibit substantial variation in root exudation and root morphology.

Apart from nutrient-scavenging strategies, root exudate-induced nutrient-mining strategies recruit beneficial microbes to the rhizosphere, which enhance nutrient availability and plant growth. Previous studies contended that P-solubilizing microbes (PSMs) have the capacity to solubilize P from recalcitrant forms of inorganic and organic P and thus enhance plant P acquisition and growth [6, 40]. Metagenomics analysis in our study further corroborated that rhizosphere microbiomes from domesticated wheat enriched more abundant inorganic P solubilization genes (e.g., *ppx* and *ppa*) via releasing of root exudates (Fig. S18). More importantly, N-cycling microbial-dominated mobilizing processes play a pivotal role in plant N acquisition and growth [5]. Metagenomics analyses based on nitrogen metabolism further support that domesticated plants selectively recruit rhizosphere keystone taxa that carry nitrogen fixation genes (e.g., *nifH*) and organic nitrogen ammonification genes (e.g., *gdhA*) to sustain plant N uptake and plant growth through the attraction of root exudates (Fig. S19). These plant-available N through microbial-dominated mobilizing processes can ultimately affect root morphological traits [1]. In vitro studies on plant-microbiome interactions, such as crop (e.g., wheat, maize, and rice) and model plants (e.g., Arabidopsis), which have provided rapid and in-depth knowledge on how the development of the root morphology traits is regulated by single or culture-dependent synthetic communities [9, 43]. To definitively explore the role of the keystone taxa in determining of plant growth-promoting features and the impacts on root architecture, analysis of associations between nutrient-cycling-related genes and root morphology traits together with in vitro experiments further highlighted that a single bacterial genus or fungi could maintain plant root growth in a complex microbiome, which is in line with a recent study [23]. Notably, we found that individual microbial consortium had inhibition effect on the root growth of wheat and thus led to poor plants. These findings suggest that although domestication-induced keystone taxa had crucial role in promoting plant growth, the ubiquitous of maleficent microbiota during plant domestication should be given more attention to develop correspondingly microbial inhibitors to sniping these harmful microbial consortiums for promoting plant growth. However, the mechanisms that how individual keystone taxa reprogramming root morphology traits needed to be explored using RNA sequencing in the further study, as well as the contribution of root exudation to colonization of individual microbial consortium at the developmental stage of wild and domesticated wheat, require more rigorous assessment in the next growing season.

**Fig. 7:**
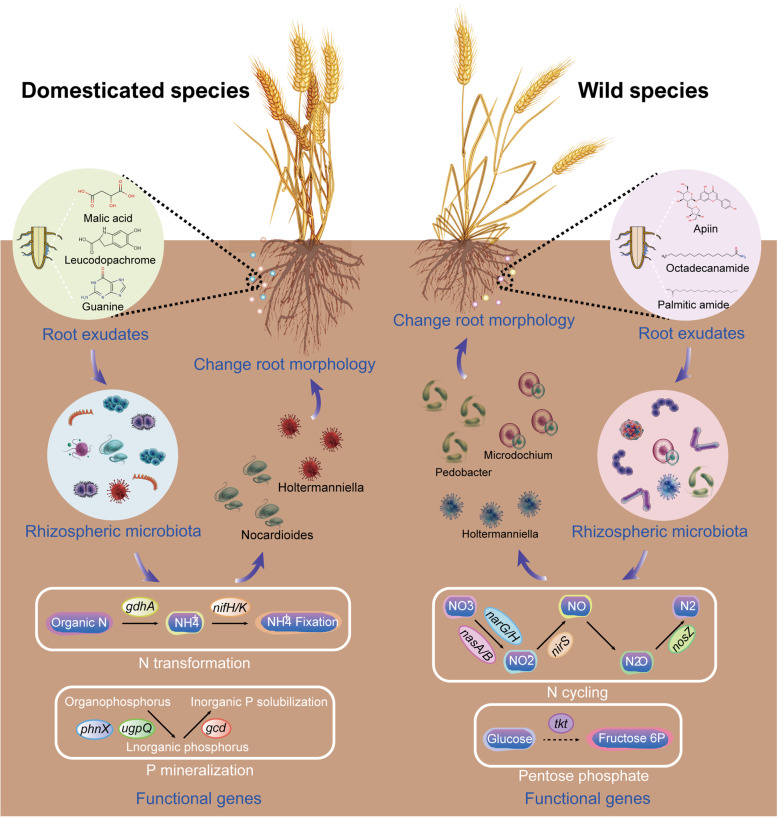
Conceptual model illustrating the plant domestication shapes rhizosphere microbiome assembly and metabolic functions

Collectively, this study provides a systematic understanding of the rhizosphere microbial communities and their metabolic functions during plant domestication processes (Fig. 7). Our findings presented here demonstrate that domestication exerted stronger selection power on the rhizosphere community assembly, although niche compartment and plant genotypes were also significant for the microbial community variations. We further demonstrated that plant domestication leads to a decline in gene diversity and a shift in microbial functional traits, particularly for functional genes related with multi-nutrients cycling. Furthermore, we found that contrasting functional roles of root exudates shaped rhizosphere microbiome and determined root morphology traits. Taken together, these findings suggest that plant domestication exerts a strong and direct selective power on the rhizosphere microbiome assembly and functional adaptation through the release of root exudates with contrasting roles. The current study significantly underpins our understanding of the species coexistence and functional adaptation of the rhizosphere microbiomes of wheat during plant domestication and paves the way for new plant breeding strategies.

## Supplementary Information

**Additional file 1:** **Fig. S1.** Alpha diversity included Shannon index, Simpson index, ACE index, and Chao index for bacteria and fungi communities in different accession of domesticated and wild wheats. Errors bars represent standard errors (*n*= 5). Different lowcase letters above the bars indicate significant differences (*P*< 0.05), based on Kruskal-Wallis test. **Fig. S2.** The Bray-Curtis dissimilarity of bacteria and fungi communities in domesticated and wild wheats. DB1~DB3 and WB1-WB3 indicates that samples are affiliated with bulk soil in the domesticated and wild wheats, respectively. DT1~DT3 and WT1-WT3 indicates that samples are affiliated with rhizosphere soil in the domesticated and wild wheats, respectively. The dots represent the values of Bray-Curtis dissimilarity between replicates in the any of groups. The details on the D1-D3 and W1-W3 groups are available in Table S2. **Fig. S3.** Constrained Analysis of Principal Coordinates analysis (CAP) ordination constraned to domestication status (left panel), habitat type (middle panel), and genome group (right panel) based on Bray-Curtis metric of bacteria and fungi communities. Variance of community dissimilarity among five treatment were draw from ANOVA-like permutation analysis. **Fig. S4.** Principal coordinate analysis ordinations (PCoA) for bacteria and fungi communities in domesticated and wild wheats. **Fig. S5.** Distribution of dominant phyla and genus in the bacteria and fungi communities in domesticated and wild wheats. **Fig. S6.** Amplicon sequences variants (ASVs) form bulk soils responsible for the community differences in the wild wheats and domesticated wheats that are calculated by a differential abundance test and random forest classification. (A) and (B) The volcano plot illustrating the enrichment and depletion patterns of rhizosphere bacterial and fungal microbiomes in the three wild wheats compared with three domesticated wheat accessions. DI, depleted index; DSI, dissimilarity index. (C) and (D) Joyplots showing the relative abundance profiles of top 20 ASVs in bacterial and fungal communities that are revealed by a Random forest (RF) classifier. The top ASVs on the genus level are listed along the y-axis represent their importance in contributing to the accuracy of domesticated and wild wheats prediction by calculating their mean decrease accuracy in the RF model. **Fig. S7.** Microbial interkingdom association networks and node-level topological features for bulk soils. (A) and (B) Interkingdom co-occurrence networks in the domesticated and wild wheats. Only compositionality-robust (p > 0.7) and statistically significant (*q* < 0.01) correlations were shown. The size of each node indicates the relative abundance of each ASV. The color of each node represents the bacteria or fungi taxa. Blue solid lines represent co-presence associations and red line represent mutual exclusive correlations. The thickness of each link line is proportional to the correlation coefficients of the connections. The keystone taxa and dominant modules for each networks were also shown. (C) and (D) Box graphs illustrating the node-level topological features of each networks, including betweenness and degree. Comparison of these two features demonstrating the high degree and low betweenness for the keystone taxa. Bar diagrams showing the proportion of inter-and intra-kingdom edges of positive or negative correlations in the rhizosphere network. The significance of differences between domesticated and wild wheats were determined by Kruskal-Wallis test. **Fig. S8.** Network modularity profiles of rhizosphere soil and bulk soil microbial communities in the domesticated and wild wheats. The eight bar plots in the left and middle columns represents microbial taxa on the class level in the module I and module II. The four bar plots in the right columns indicates the proportion of ASVs that affiliated with bacteria and fungi communities in the module I and module II. **Fig. S9.** The volcano plot illustrating the enrichment and depletion patterns of KO functional categories and KO pathway in the wild wheats compared with domesticated wheat accessions. The KOs were colored by their categorization as “wild-enriched”, “domesticated-enriched”, and “non-differential” according to their values of Log2(count per million) and Log2(fold change). **Fig. S10.** (A) Nonmetric multidimensional scaling (NMDS) ordination of KEGG Orthology, CAZyome, and COG based on Bray-Curtis distances. The significance of differences between domesticated and wild wheats were determined by Kruskal-Wallis test. (B) Heat map illustrating the relative abundance (Z-score) of functional genes (based on KO) affiliated with carbon cycling in different accession of wheats. **Fig. S11.** Heat map illustrating the relative abundance (Z-score) of functional genes (based on KO) affiliated with nitrogen (A) and phosphorus (B) cycling in different accession of wheats. **Fig. S12.** (A-H) Random forest (RF) mean predictor importance of KO functional categories as drivers for the plant phenotypes, including (A) plant height (PH), (B) chlorophyll content (CC), (C) ear length (EL), (D) subsegment length (SSL), (E) Tiller, (F) thousand grain weight (TGW), (G) seed length (SL), and (H) seed width (SW), respectively. The accuracy importance measure was calculated for each tree and averaged over the forest (2000 trees). Percentage increase in the mean squared error (MSE) of variables were applied to evaluate the importance of these predictors, and higher MSE% values represent more important predictors. Significance levels of each predictor are as follows: *P< 0.05, **P< 0.01, and ***P< 0.001. **Fig. S13.** Partial least squares discriminant analysis (PLS-DA) and orthogonal partial least squares discriminant analysis (OPLS-DA) for root exudates that extracted from roots of wild and domesticated wheats. **Fig. S14.** (A) The average relative abundance of enriched root exudates in the wild and domesticated wheats. (B) The fold changes of enriched metabolites in domesticated and wild wheats. The error bars represent standard errors of sample replicates and asterisks (*) indicate metabolic categories that are significantly more predominant in wild or domesticated wheats (P value <0.05, Wilcoxon test). (C) Heat map illustrating the relative abundance of root exudates in different accession of wheats. **Fig. S15.** (A-H) Random forest (RF) mean predictor importance of enriched metabolites as drivers for the plant phenotypes, including (A) plant height (PH), (B) chlorophyll content (CC), (C) ear length (EL), (D) subsegment length (SSL), (E) Tiller, (F) thousand grain weight (TGW), (G) seed length (SL), and (H) seed width (SW), respectively. The accuracy importance measure was calculated for each tree and averaged over the forest (2000 trees). Percentage increase in the mean squared error (MSE) of variables were applied to evaluate the importance of these predictors, and higher MSE% values represent more important predictors. Significance levels of each predictor are as follows: *P< 0.05, **P< 0.01, and ***P< 0.001. **Fig. S16.** (A-D) Ecological associations between root morphology and rhizosphere bacteria and fungi in domesticated wheat and wild wheats. Root morphology traits including root volume (RV), root average diameter (RAD), stem thickness (ST), root length (RL), fresh weight of root (FWR), were scanned and registered using Microtek ScanMaker i800 plus system. Significance levels of each association are as follows: *P< 0.05, **P< 0.01, and ***P< 0.001. **Fig. S17.** (A) Pearson correlation relationships between edaphic variables and carbon-cycling-related functional gene. (B) Pearson correlation relationships between dominant metabolites and carbon-cycling-related functional gene. Significance levels of each association are as follows: *P< 0.05, **P< 0.01, and ***P< 0.001. **Fig. S18.** Pearson correlation relationships between dominant metabolites and phosphorus-cycling-related functional gene. Significance levels of each association are as follows: *P< 0.05, **P< 0.01, and ***P< 0.001. **Fig. S19.** Pearson correlation relationship between dominant metabolites and nitrogen-cycling-related functional gene. Significance levels of each association are as follows: *P< 0.05, **P< 0.01, and ***P< 0.001.

**Additional file 2: Table S1.** Plant phenotypes of 44 Tetraploid wheat species after nine years cultivation. **Table S2.** Plant phenotypes of six selected Tetraploid wheat species, involving T. turgidum Espana (labelled “D1”), T. turgidum Mexicanos (labelled “D2”), T. turgidum Kazakhstan (labelled “D3”), T. turgidum Turkey III (labelled “W1”), T. turgidum Syria VI (labelled “W2”), and T. turgidum Jordan III (labelled “W3”). Five replicates of each wheat accessions were randomly chosen to measure the plant attributes, including plant height, ear length, sub-segment length, and tiller. **Table S3.** Effect of domestication status, genome type and niche compartment on the bacterial and fungal communities based on PERMANOVA analysis. **Table S4.** Analysis of ANOSIM testing microbial communities from rhizosphere and bulk soils based on the Bray-Curtis across different treatments. Significance levels of each predictor are as follows: *P< 0.05, **P< 0.01, and ***P< 0.001. **Table S5.** Topological features of microbial interkingdom association networks in different domesticated status and their corresponding random networks. **Table S6.** Characteristics of metagenomics sequencing of rhizosphere microbiomes in wild and domesticated wheats. **Table S7.** Relative abundance of the metagenomics microbial function profiling (KEGG Orthology function category) in wild and domesticated wheats. **Table S8.** Root morphology trait of wild and domesticated wheat grown in Hogland nutrient solution and inoculation with rhizosphere microbiota. **Table S9.** Characteristics of soil samples in different plots for domesticated and whild wheats. Soil nutrients include total carbon (TC), total nitrogen (TN), total phosphorus (TP), total organic carbon (TOC), dissolved organic carbon (DOC), dissolved organic nitrogen (DON), nitrate (NO3-), ammonium (NH4+), soil available phosphorus (AP), soil available potassium (AK).

**Additional file 3:** **Method S1.** Experiment design and field site management. **Method S2.** Samples collection, DNA extraction, PCR amplification and amplicon sequencing.** Method S3.** Shotgun metagenomics sequencing. **Method S4.** Root exudate collection and LC-MS analysis. **Method S5.** Microbiota inoculation experiment. **Method S6.** Statistical analyses.

## Data Availability

The core codes that support the findings of this study have been deposited in the GitHub (https://github.com/Duntao/Project_of_Domestication). The raw sequences have been deposited in the National Center for Biotechnology Information (NCBI) database with accession number PRJNA766139 for amplicon sequencing and PRJNA766164 for metagenomics sequences.
