# Supplementary material for: Plant domestication shapes rhizosphere microbiome assembly and metabolic functions
Source: Microbiome. 2023 Mar 31;11:70. doi: 10.1186/s40168-023-01513-1 (PMC10064753; doi:10.1186/s40168-023-01513-1)

**Supporting Information**

**Plant domestication shapes rhizosphere microbiome assembly and metabolic functions**

Hong Yue^1#^, Wenjie Yue^1#^, Shuo Jiao^2,3^, Hyun Kim^4^, Yong-Hwan Lee^4^,

Gehong Wei^2,3 ***^, Weining Song^1 **^, Duntao Shu^2,3 *^

^1^ College of Agronomy, State Key Laboratory of Crop Stress Biology in Arid Areas, Northwest A&F University, Yangling, Shaanxi 712100, China

^2^ Shaanxi Key Laboratory of Agricultural and Environmental Microbiology, Yangling, Shaanxi 712100, China

^3^ State Key Laboratory of Crop Stress Biology in Arid Areas, College of Life Sciences, Northwest A&F University, Yangling, Shaanxi 712100, China

^4^ Department of Agricultural Biotechnology, Seoul National University, Seoul 08826, Korea

^#^ These authors contributed equally to this work

^*^ Corresponding author: [donald.shu@nwafu.edu.cn](mailto:donald.shu@nwafu.edu.cn) (D. Shu)

^**^ Corresponding author: sweining2002@yahoo.com (W. Song)

^***^ Corresponding author: [weigehong@nwafu.edu.cn](mailto:weigehong@nwafu.edu.cn) (G. Wei)

Tel: 86-18710994335; Fax: 86-29-87090162

**Contents**

**1. Tables**

**Table S1** Plant phenotypes of 44 Tetraploid wheat species after nine years cultivation.

**Table S2** Plant phenotypes of six selected Tetraploid wheat species, involving *T. turgidum Espana* (labelled “D1”), *T. turgidum Mexicanos* (labelled “D2”), *T. turgidum Kazakhstan* (labelled “D3”), *T. turgidum Turkey III* (labelled “W1”), *T. turgidum Syria VI* (labelled “W2”), and *T. turgidum Jordan III* (labelled “W3”). Five replicates of each wheat accessions were randomly chosen to measure the plant attributes, including plant height, ear length, sub-segment length, and tiller.

**Table S3** Effect of domestication status, genome type and niche compartment on the bacterial and fungal communities based on PERMANOVA analysis.

**Table S4** Analysis of ANOSIM testing microbial communities from rhizosphere and bulk soils based on the Bray-Curtis across different treatments. Significance levels of each predictor are as follows: **P*< 0.05, ***P*< 0.01, and ****P*< 0.001.

**Table S5** Topological features of microbial interkingdom association networks in different domesticated status and their corresponding random networks

**Table S6** Characteristics of metagenomics sequencing of rhizosphere microbiomes in wild and domesticated wheats.

**Table S7** Relative abundance of the metagenomics microbial function profiling (KEGG Orthology function category) in wild and domesticated wheats.

**Table S8** Root morphology trait of wild and domesticated wheat grown in Hogland nutrient solution and inoculation with rhizosphere microbiota.

**Table S9** Characteristics of soil samples in different plots for domesticated and whild wheats. Soil nutrients include total carbon (TC), total nitrogen (TN), total phosphorus (TP), total organic carbon (TOC), dissolved organic carbon (DOC), dissolved organic nitrogen (DON), nitrate (NO_3_^-^), ammonium (NH_4_^+^), soil available phosphorus (AP), soil available potassium (AK).

**2. Figures**

**Fig. S1** Alpha diversity included Shannon index, Simpson index, ACE index, and Chao index for bacteria and fungi communities in different accession of domesticated and wild wheats. Errors bars represent standard errors (n= 5). Different lowcase letters above the bars indicate significant differences (*P*< 0.05), based on Kruskal-Wallis test.

**Fig. S2** The Bray-Curtis dissimilarity of bacteria and fungi communities in domesticated and wild wheats. DB1~DB3 and WB1-WB3 indicates that samples are affiliated with bulk soil in the domesticated and wild wheats, respectively. DT1~DT3 and WT1-WT3 indicates that samples are affiliated with rhizosphere soil in the domesticated and wild wheats, respectively. The dots represent the values of Bray-Curtis dissimilarity between replicates in the any of groups. The details on the D1-D3 and W1-W3 groups are available in Table S2.

**Fig. S3** Constrained Analysis of Principal Coordinates analysis (CAP) ordination constraned to domestication status (left panel), habitat type (middle panel), and genome group (right panel) based on Bray-Curtis metric of bacteria and fungi communities. Variance of community dissimilarity among five treatment were draw from ANOVA-like permutation analysis.

**Fig. S4** Principal coordinate analysis ordinations (PCoA) for bacteria and fungi communities in domesticated and wild wheats.

**Fig. S5** Distribution of dominant phyla and genus in the bacteria and fungi communities in domesticated and wild wheats.

**Fig. S6** Amplicon sequences variants (ASVs) form bulk soils responsible for the community differences in the wild wheats and domesticated wheats that are calculated by a differential abundance test and random forest classification. (A) and (B) The volcano plot illustrating the enrichment and depletion patterns of rhizosphere bacterial and fungal microbiomes in the three wild wheats compared with three domesticated wheat accessions. DI, depleted index; DSI, dissimilarity index. (C) and (D) Joyplots showing the relative abundance profiles of top 20 ASVs in bacterial and fungal communities that are revealed by a Random forest (RF) classifier. The top ASVs on the genus level are listed along the y-axis represent their importance in contributing to the accuracy of domesticated and wild wheats prediction by calculating their mean decrease accuracy in the RF model.

**Fig. S7** Microbial interkingdom association networks and node-level topological features for bulk soils. (A) and (B) Interkingdom co-occurrence networks in the domesticated and wild wheats. Only compositionality-robust (|*ρ*|> 0.7) and statistically significant (*q* < 0.01) correlations were shown. The size of each node indicates the relative abundance of each ASV. The color of each node represents the bacteria or fungi taxa. Blue solid lines represent co-presence associations and red line represent mutual exclusive correlations. The thickness of each link line is proportional to the correlation coefficients of the connections. The keystone taxa and dominant modules for each networks were also shown. (C) and (D) Box graphs illustrating the node-level topological features of each networks, including betweenness and degree. Comparison of these two features demonstrating the high degree and low betweenness for the keystone taxa. Bar diagrams showing the proportion of inter-and intra-kingdom edges of positive or negative correlations in the rhizosphere network. The significance of differences between domesticated and wild wheats were determined by Kruskal-Wallis test.

**Fig. S8** Network modularity profiles of rhizosphere soil and bulk soil microbial communities in the domesticated and wild wheats. The eight bar plots in the left and middle columns represents microbial taxa on the class level in the module I and module II. The four bar plots in the right columns indicates the proportion of ASVs that affiliated with bacteria and fungi communities in the module I and module II.

**Fig. S9** The volcano plot illustrating the enrichment and depletion patterns of KO functional categories and KO pathway in the wild wheats compared with domesticated wheat accessions. The KOs were colored by their categorization as “wild-enriched”, “domesticated-enriched”, and “non-differential” according to their values of Log_2_(count per million) and Log_2_(fold change).

**Fig. S10** (A) Nonmetric multidimensional scaling (NMDS) ordination of KEGG Orthology, CAZyome, and COG based on Bray-Curtis distances. The significance of differences between domesticated and wild wheats were determined by Kruskal-Wallis test. (B) Heat map illustrating the relative abundance (Z-score) of functional genes (based on KO) affiliated with carbon cycling in different accession of wheats.

**Fig. S11** Heat map illustrating the relative abundance (Z-score) of functional genes (based on KO) affiliated with nitrogen (A) and phosphorus (B) cycling in different accession of wheats.

**Fig. S12** (A-H) Random forest (RF) mean predictor importance of KO functional categories as drivers for the plant phenotypes, including (A) plant height (PH), (B) chlorophyll content (CC), (C) ear length (EL), (D) subsegment length (SSL), (E) Tiller, (F) thousand grain weight (TGW), (G) seed length (SL), and (H) seed width (SW), respectively. The accuracy importance measure was calculated for each tree and averaged over the forest (2000 trees). Percentage increase in the mean squared error (MSE) of variables were applied to evaluate the importance of these predictors, and higher MSE% values represent more important predictors. Significance levels of each predictor are as follows: **P*< 0.05, ***P*< 0.01, and ****P*< 0.001.

**Fig. S13** Partial least squares discriminant analysis (PLS-DA) and orthogonal partial least squares discriminant analysis (OPLS-DA) for root exudates that extracted from roots of wild and domesticated wheats.

**Fig. S14** (A) The average relative abundance of enriched root exudates in the wild and domesticated wheats. (B) The fold changes of enriched metabolites in domesticated and wild wheats. The error bars represent standard errors of sample replicates and asterisks (*) indicate metabolic categories that are significantly more predominant in wild or domesticated wheats (*P* value <0.05, Wilcoxon test). (C) Heat map illustrating the relative abundance of root exudates in different accession of wheats.

**Fig. S15** (A-H) Random forest (RF) mean predictor importance of enriched metabolites as drivers for the plant phenotypes, including (A) plant height (PH), (B) chlorophyll content (CC), (C) ear length (EL), (D) subsegment length (SSL), (E) Tiller, (F) thousand grain weight (TGW), (G) seed length (SL), and (H) seed width (SW), respectively. The accuracy importance measure was calculated for each tree and averaged over the forest (2000 trees). Percentage increase in the mean squared error (MSE) of variables were applied to evaluate the importance of these predictors, and higher MSE% values represent more important predictors. Significance levels of each predictor are as follows: **P*< 0.05, ***P*< 0.01, and ****P*< 0.001.

**Fig. S16** (A-D) Ecological associations between root morphology and rhizosphere bacteria and fungi in domesticated wheat and wild wheats. Root morphology traits including root volume (RV), root average diameter (RAD), stem thickness (ST), root length (RL), fresh weight of root (FWR), were scanned and registered using Microtek ScanMaker i800 plus system. Significance levels of each association are as follows: **P*< 0.05, ***P*< 0.01, and ****P*< 0.001.

**Fig. S17** (A) Pearson correlation relationships between edaphic variables and carbon-cycling-related functional gene. (B) Pearson correlation relationships between dominant metabolites and carbon-cycling-related functional gene. Significance levels of each association are as follows: **P*< 0.05, ***P*< 0.01, and ****P*< 0.001.

**Fig. S18** Pearson correlation relationships between dominant metabolites and phosphorus-cycling-related functional gene. Significance levels of each association are as follows: **P*< 0.05, ***P*< 0.01, and ****P*< 0.001.

**Fig. S19** Pearson correlation relationships between dominant metabolites and nitrogen-cycling-related functional gene. Significance levels of each association are as follows: **P*< 0.05, ***P*< 0.01, and ****P*< 0.001.

**Table S1** Plant phenotypes of 44 Tetraploid wheat species after nine years cultivation.

See the **Additional file 1**: Table S1 (XLSX).

**Table S2** Plant phenotypes of six selected Tetraploid wheat species, involving *T. turgidum Espana* (labelled “D1”), *T. turgidum Mexicanos* (labelled “D2”), *T. turgidum Kazakhstan* (labelled “D3”), *T. turgidum Turkey III* (labelled “W1”), *T. turgidum Syria VI* (labelled “W2”), and *T. turgidum Jordan III* (labelled “W3”). Five replicates of each wheat accessions were randomly chosen to measure the plant attributes, including plant height, ear length, sub-segment length, and tiller. See the **Additional file 1**: Table S2 (XLSX).

**Table S3** Effect of domestication status, genome type and niche compartment on the bacterial and fungal communities based on PERMANOVA analysis.

See the **Additional file 1**: Table S3 (XLSX).

**Table S4** Analysis of ANOSIM testing microbial communities from rhizosphere and bulk soils based on the Bray-Curtis across different treatments. Significance levels of each predictor are as follows: **P*< 0.05, ***P*< 0.01, and ****P*< 0.001.

See the **Additional file 1**: Table S4 (XLSX).

**Table S5** Topological features of microbial interkingdom association networks in different domesticated status and their corresponding random networks

See the **Additional file 1**: Table S5 (XLSX).

**Table S6** Characteristics of metagenomics sequencing of rhizosphere microbiomes in wild and domesticated wheats.

See the **Additional file 1**: Table S6 (XLSX).

**Table S7** Relative abundance of the metagenomics microbial function profiling (KEGG Orthology function category) in wild and domesticated wheats.

See the **Additional file 1**: Table S7 (XLSX).

**Table S8** Root morphology trait of wild and domesticated wheat grown in Hogaland nutrient solution and inoculation with rhizosphere microbiota.

See the **Additional file 1**: Table S8 (XLSX).

**Table S9** Characteristics of soil samples in different plots for domesticated and whild wheats. Total carbon (TC), total nitrogen (TN), total phosphorus (TP), total organic carbon (TOC), dissolved organic carbon (DOC) and nitrogen (DON), nitrate (NO_3_^-^), ammonium (NH_4_^+^), available phosphorus (AP) and potassium (AK). See the **Additional file 1**: Table S9 (XLSX).

**Fig. S1** Alpha diversity included Shannon index, Simpson index, ACE index, and Chao index for bacteria and fungi communities in different accession of domesticated and wild wheats. Errors bars represent standard errors (n= 5). Different lowcase letters above the bars indicate significant differences (*P*< 0.05), based on Kruskal-Wallis test.


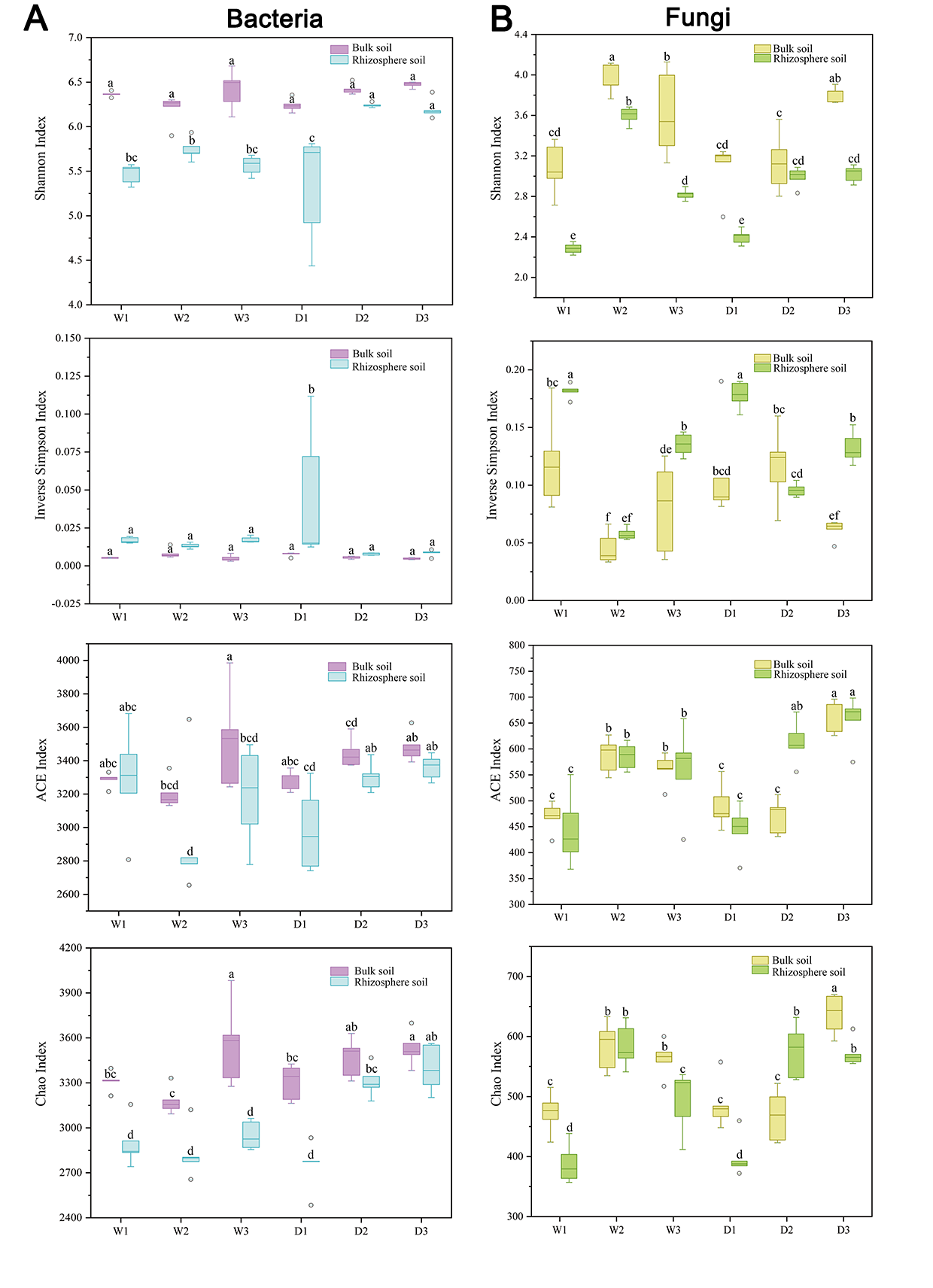


**Fig. S2** The Bray-Curtis dissimilarity of bacteria and fungi communities in domesticated and wild wheats. DB1~DB3 and WB1-WB3 indicates that samples are affiliated with bulk soil in the domesticated and wild wheats, respectively. DT1~DT3 and WT1-WT3 indicates that samples are affiliated with rhizosphere soil in the domesticated and wild wheats, respectively. The dots represent the values of Bray-Curtis dissimilarity between replicates in the any of groups. The details on the D1-D3 and W1-W3 groups are available in Table S2.


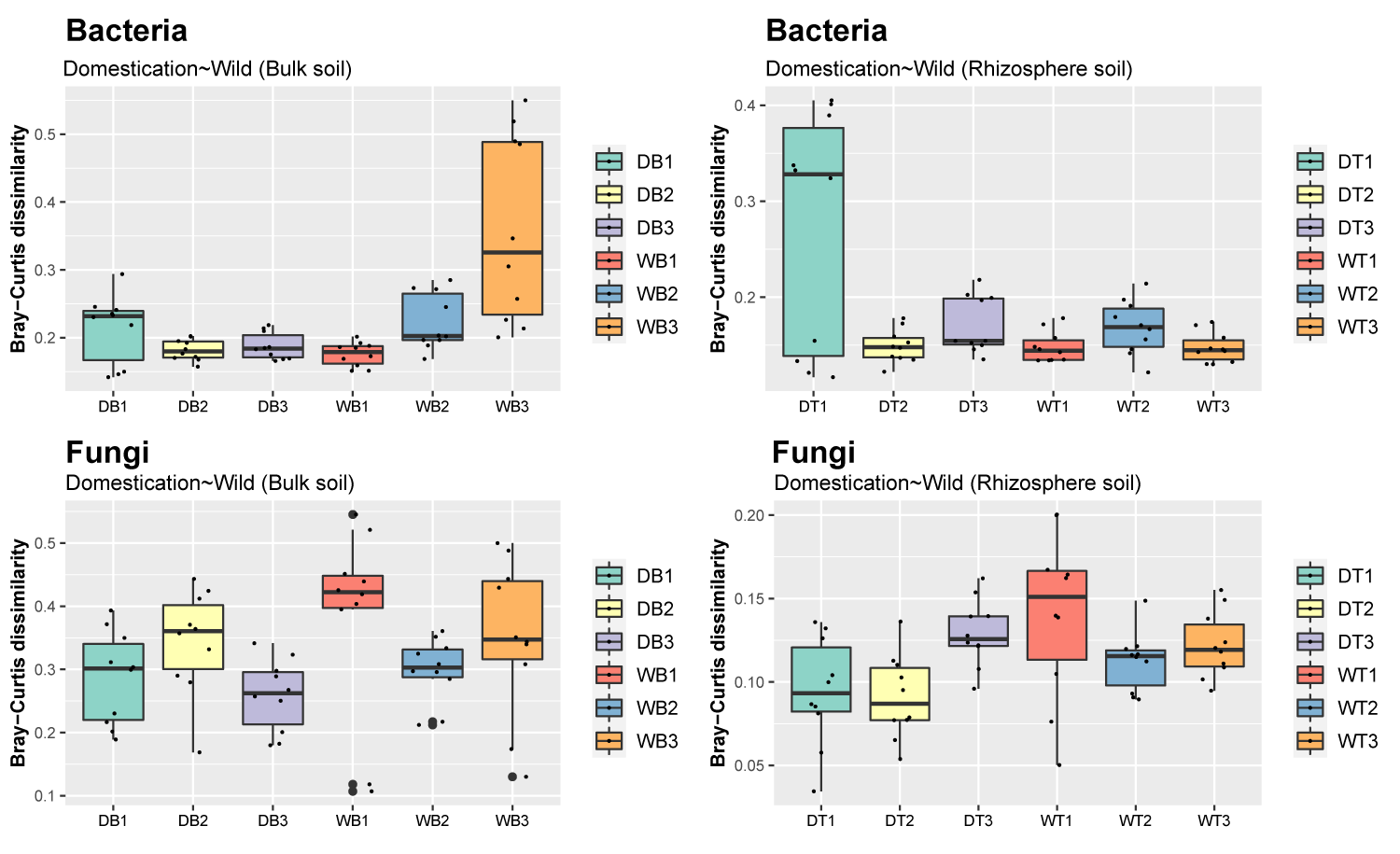


**Fig. S3** Constrained Analysis of Principal Coordinates analysis (CAP) ordination constraned to domestication status (left panel), habitat type (middle panel), and genome group (right panel) based on Bray-Curtis metric of bacteria and fungi communities. Variance of community dissimilarity among five treatment were draw from ANOVA-like permutation analysis.


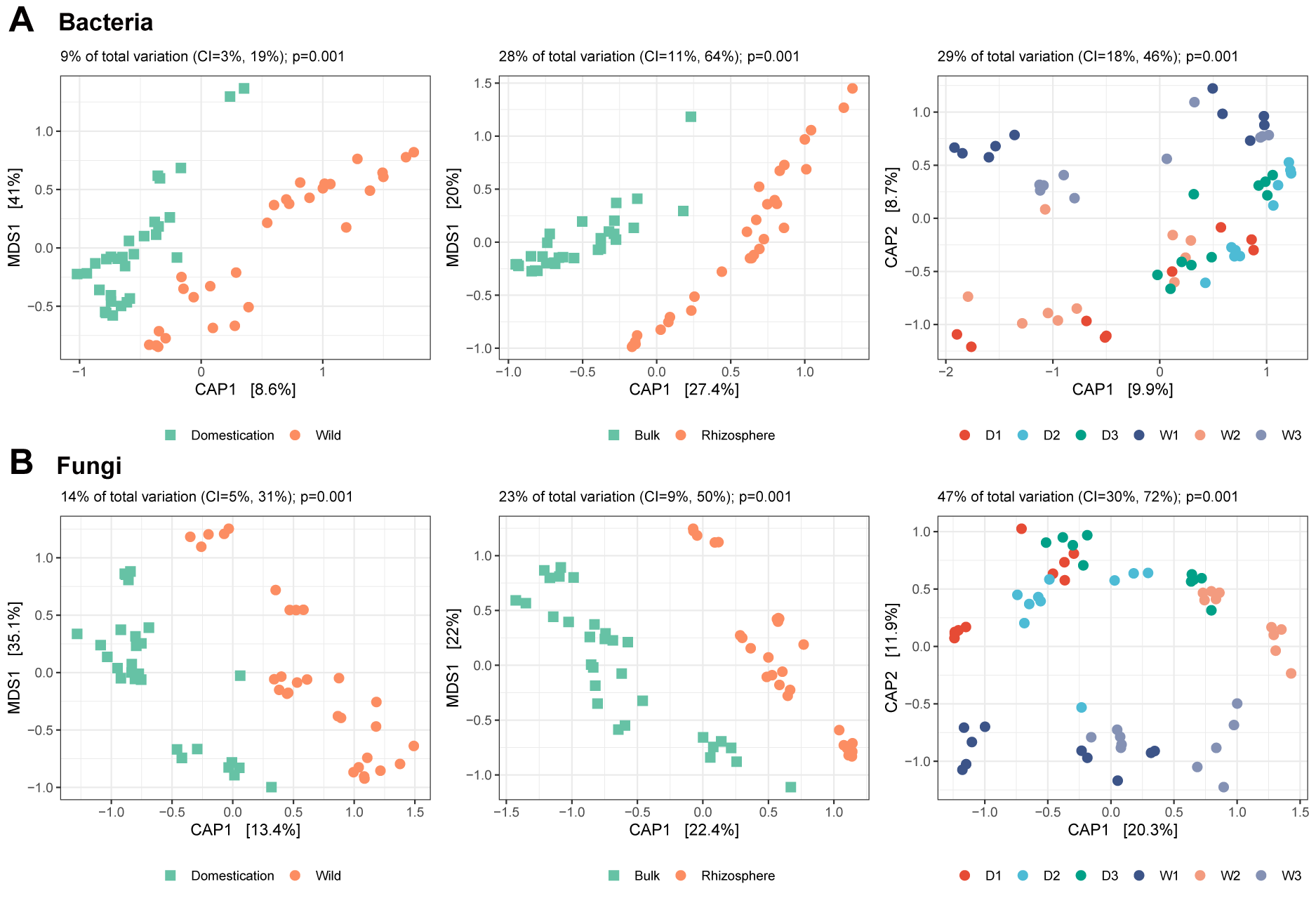


**Fig. S4** Principal coordinate analysis ordinations (PCoA) for bacteria and fungi communities in domesticated and wild wheats.

**
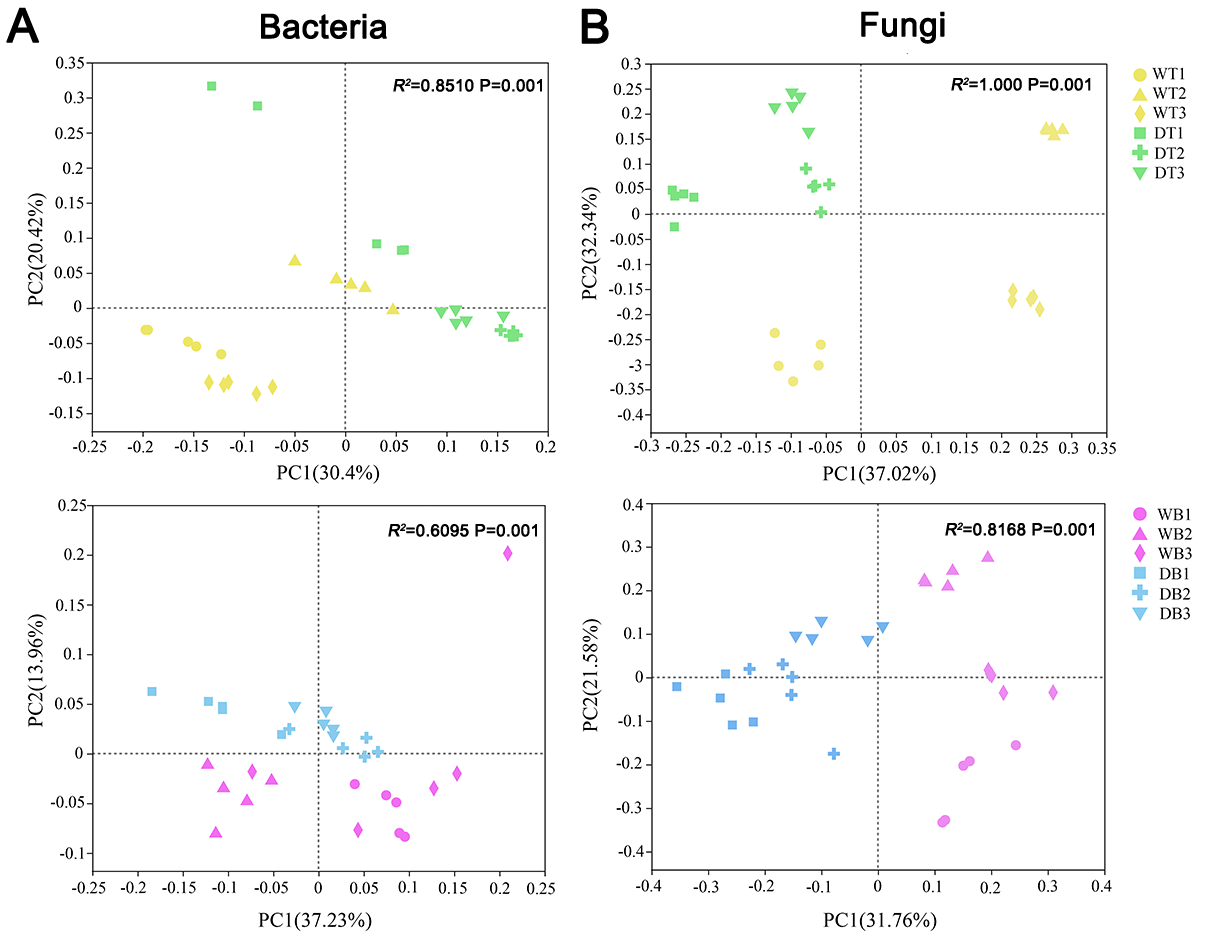
**

**Fig. S5** Distribution of dominant phyla and genus in the bacteria and fungi communities in domesticated and wild wheats.

**
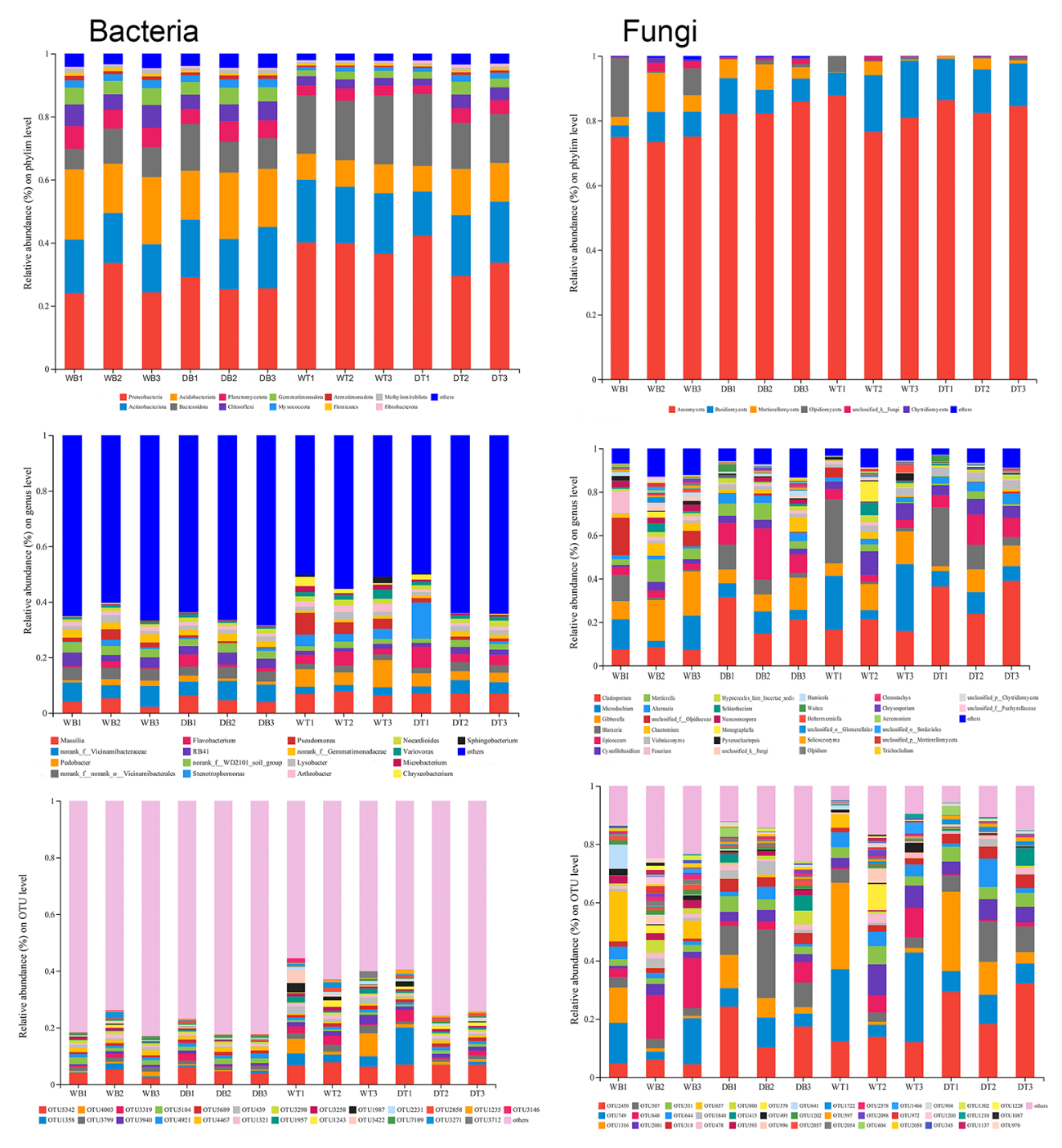
**

**Fig. S6** Amplicon sequences variants (ASVs) form bulk soils responsible for the community differences in the wild wheats and domesticated wheats that are calculated by a differential abundance test and random forest classification. (A) and (B) The volcano plot illustrating the enrichment and depletion patterns of rhizosphere bacterial and fungal microbiomes in the three wild wheats compared with three domesticated wheat accessions. DI, depleted index; DSI, dissimilarity index. (C) and (D) Joyplots showing the relative abundance profiles of top 20 ASVs in bacterial and fungal communities that are revealed by a Random forest (RF) classifier. The top ASVs on the genus level are listed along the y-axis represent their importance in contributing to the accuracy of domesticated and wild wheats prediction by calculating their mean decrease accuracy in the RF model.

**
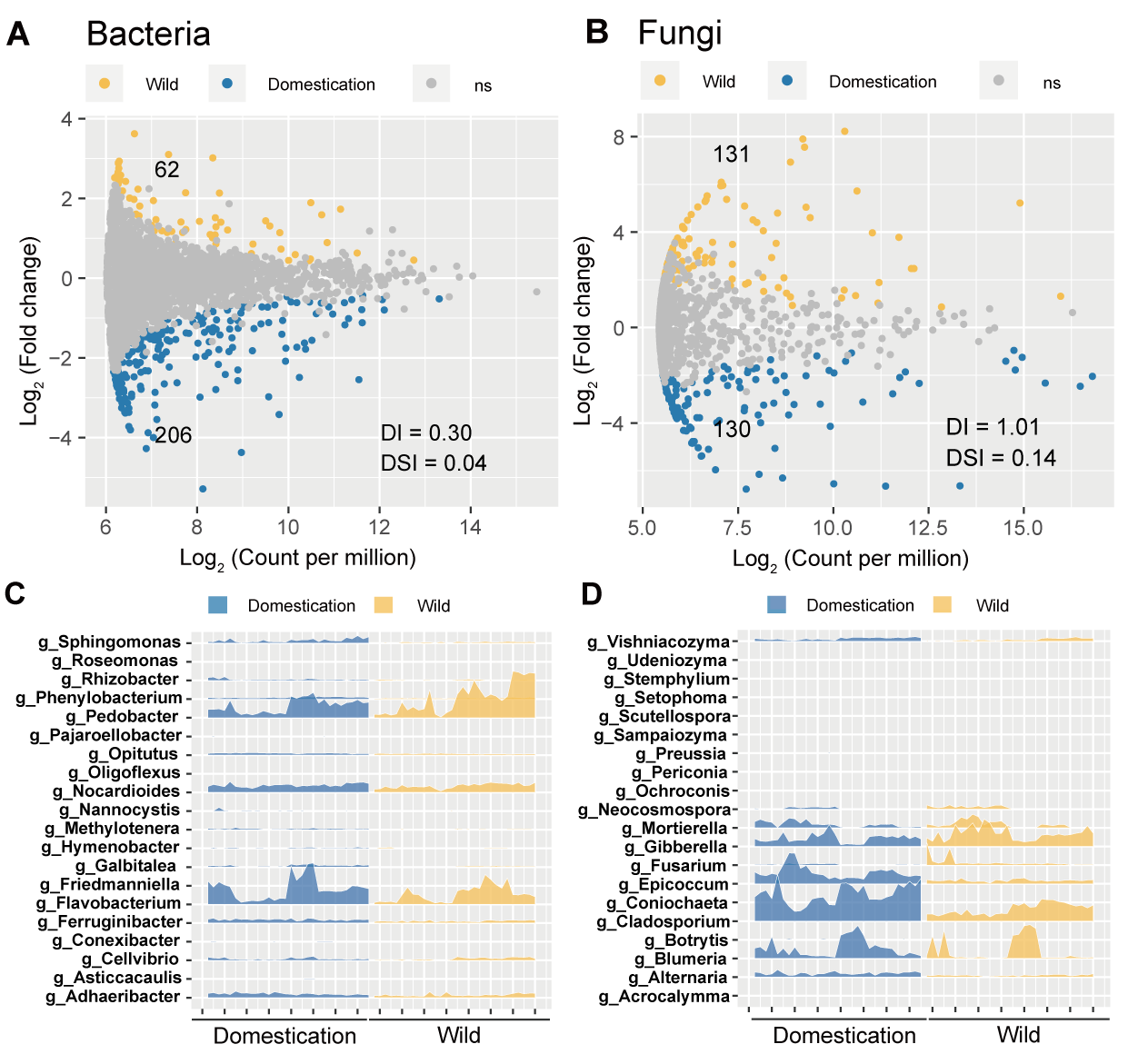
**

**Fig. S7** Microbial interkingdom association networks and node-level topological features for bulk soils. (A) and (B) Interkingdom co-occurrence networks in the domesticated and wild wheats. Only compositionality-robust (|*ρ*|> 0.7) and statistically significant (*q* < 0.01) correlations were shown. The size of each node indicates the relative abundance of each ASV. The color of each node represents the bacteria or fungi taxa. Blue solid lines represent co-presence associations and red line represent mutual exclusive correlations. The thickness of each link line is proportional to the correlation coefficients of the connections. The keystone taxa and dominant modules for each networks were also shown. (C) and (D) Box graphs illustrating the node-level topological features of each networks, including betweenness and degree. Comparison of these two features demonstrating the high degree and low betweenness for the keystone taxa. Bar diagrams showing the proportion of inter-and intra-kingdom edges of positive or negative correlations in the rhizosphere network. The significance of differences between domesticated and wild wheats were determined by Kruskal-Wallis test.


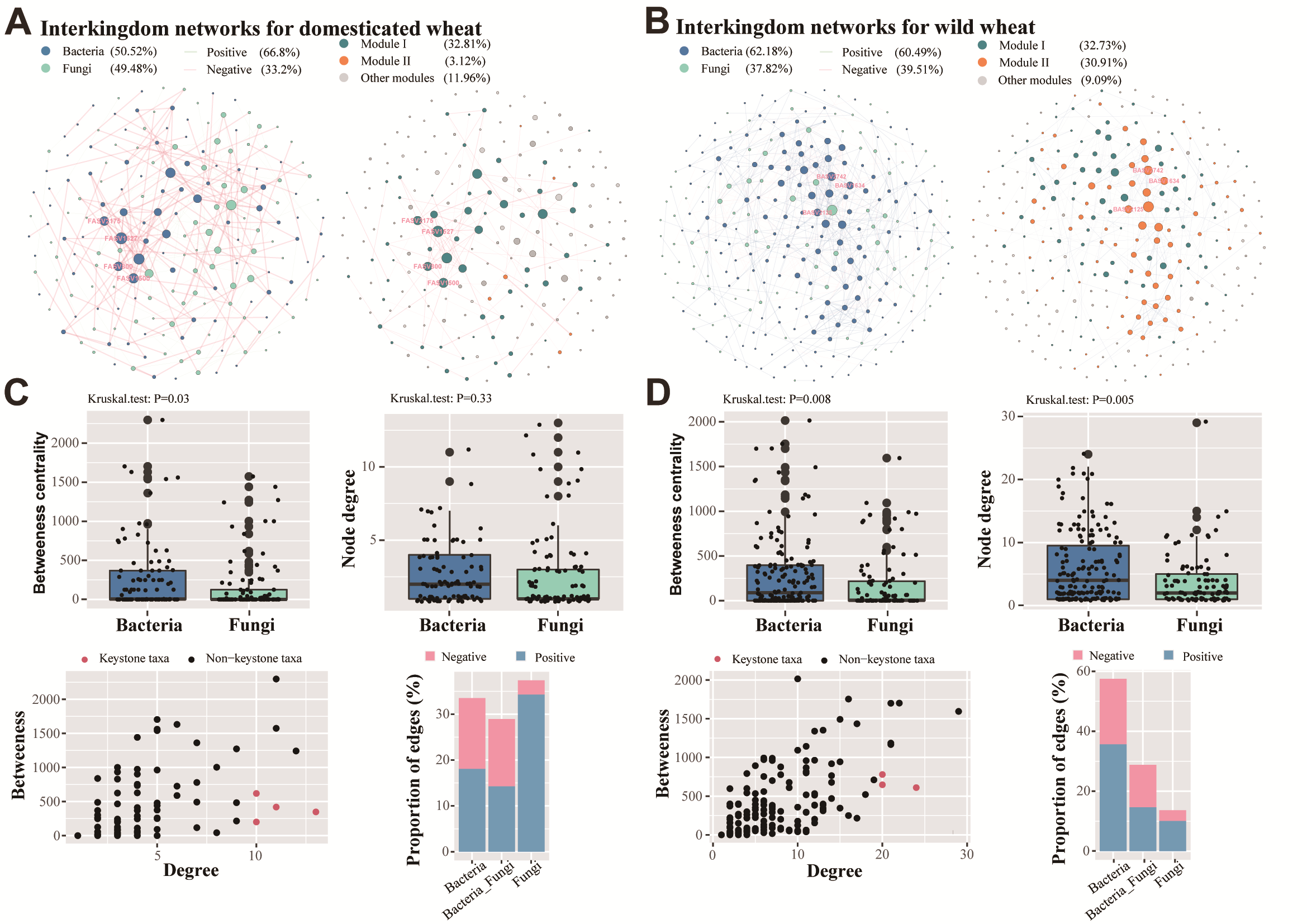


**Fig. S8** Network modularity profiles of rhizosphere soil and bulk soil microbial communities in the domesticated and wild wheats. The eight bar plots in the left and middle columns represents microbial taxa on the class level in the module I and module II. The four bar plots in the right columns indicates the proportion of ASVs that affiliated with bacteria and fungi communities in the module I and module II.


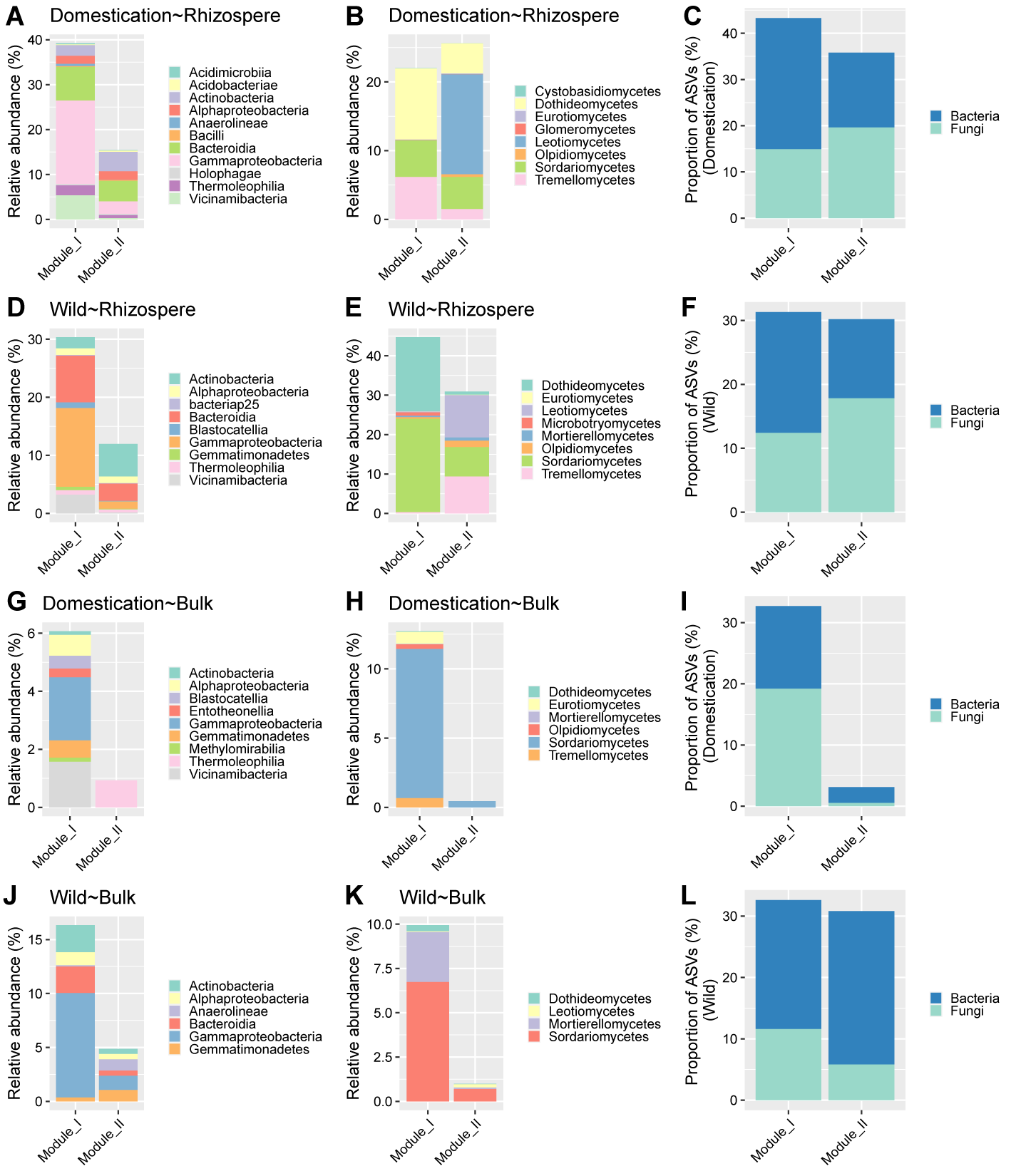


**Fig. S9** The volcano plot illustrating the enrichment and depletion patterns of KO functional categories and KO pathway in the wild wheats compared with domesticated wheat accessions. The KOs were colored by their categorization as “wild-enriched”, “domesticated-enriched”, and “non-differential” according to their values of Log_2_(count per million) and Log_2_(fold change).


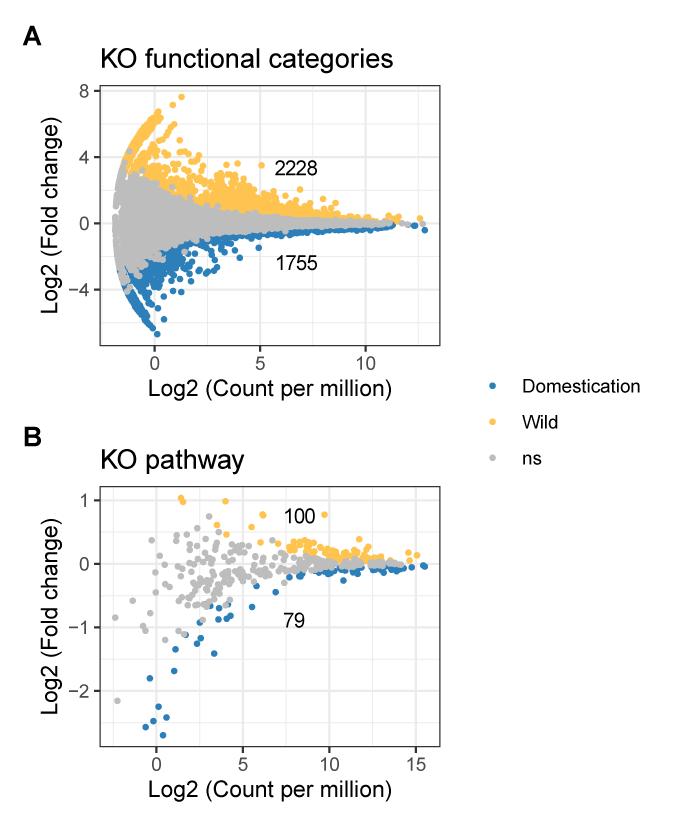


**Fig. S10** (A) Nonmetric multidimensional scaling (NMDS) ordination of KEGG Orthology, CAZyome, and COG based on Bray-Curtis distances. The significance of differences between domesticated and wild wheats were determined by Kruskal-Wallis test. (B) Heat map illustrating the relative abundance (Z-score) of functional genes (based on KO) affiliated with carbon cycling in different accession of wheats.


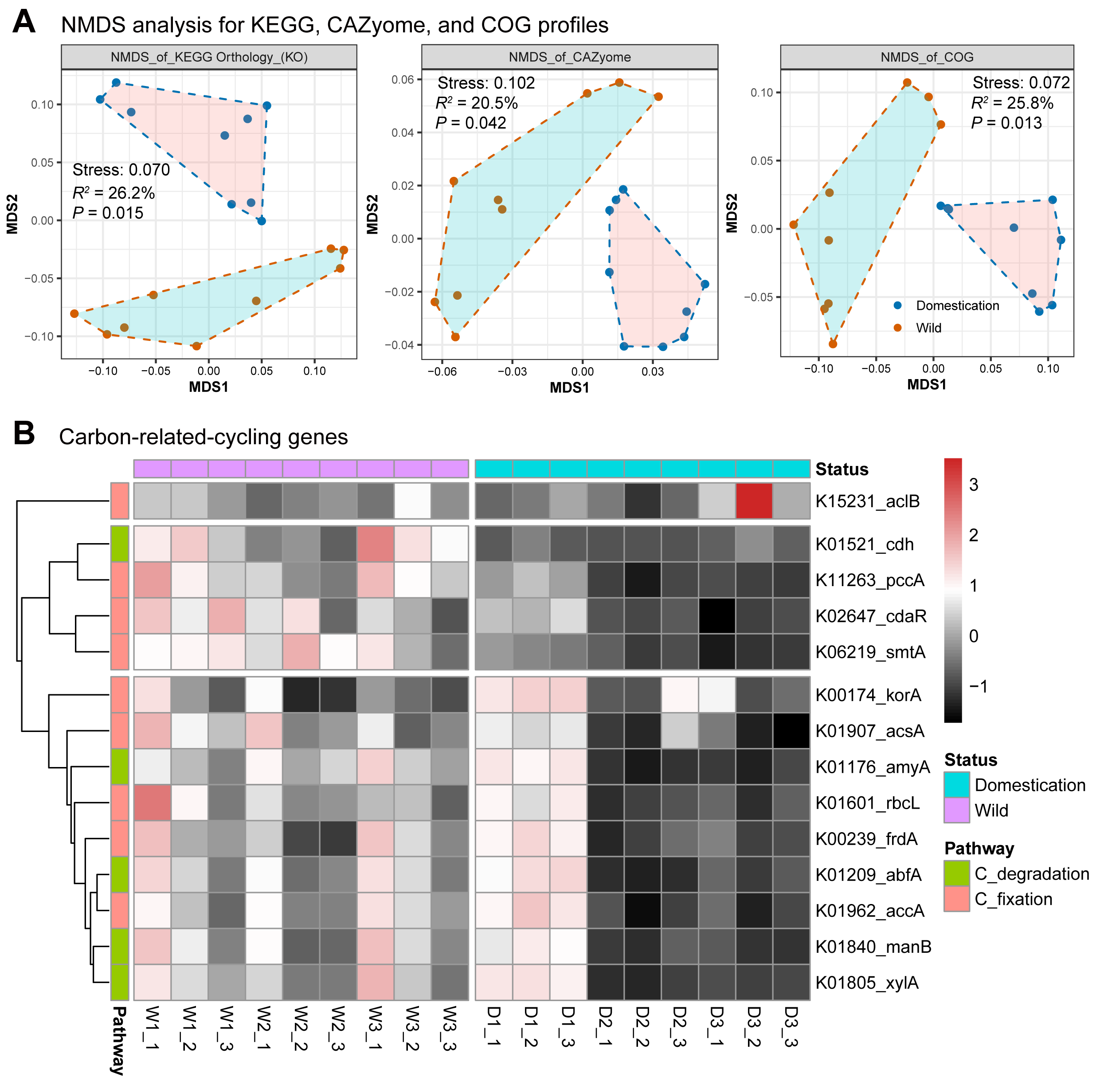


**Fig. S11** Heat map illustrating the relative abundance (Z-score) of functional genes (based on KO) affiliated with nitrogen (A) and phosphorus (B) cycling in different accession of wheats.

**
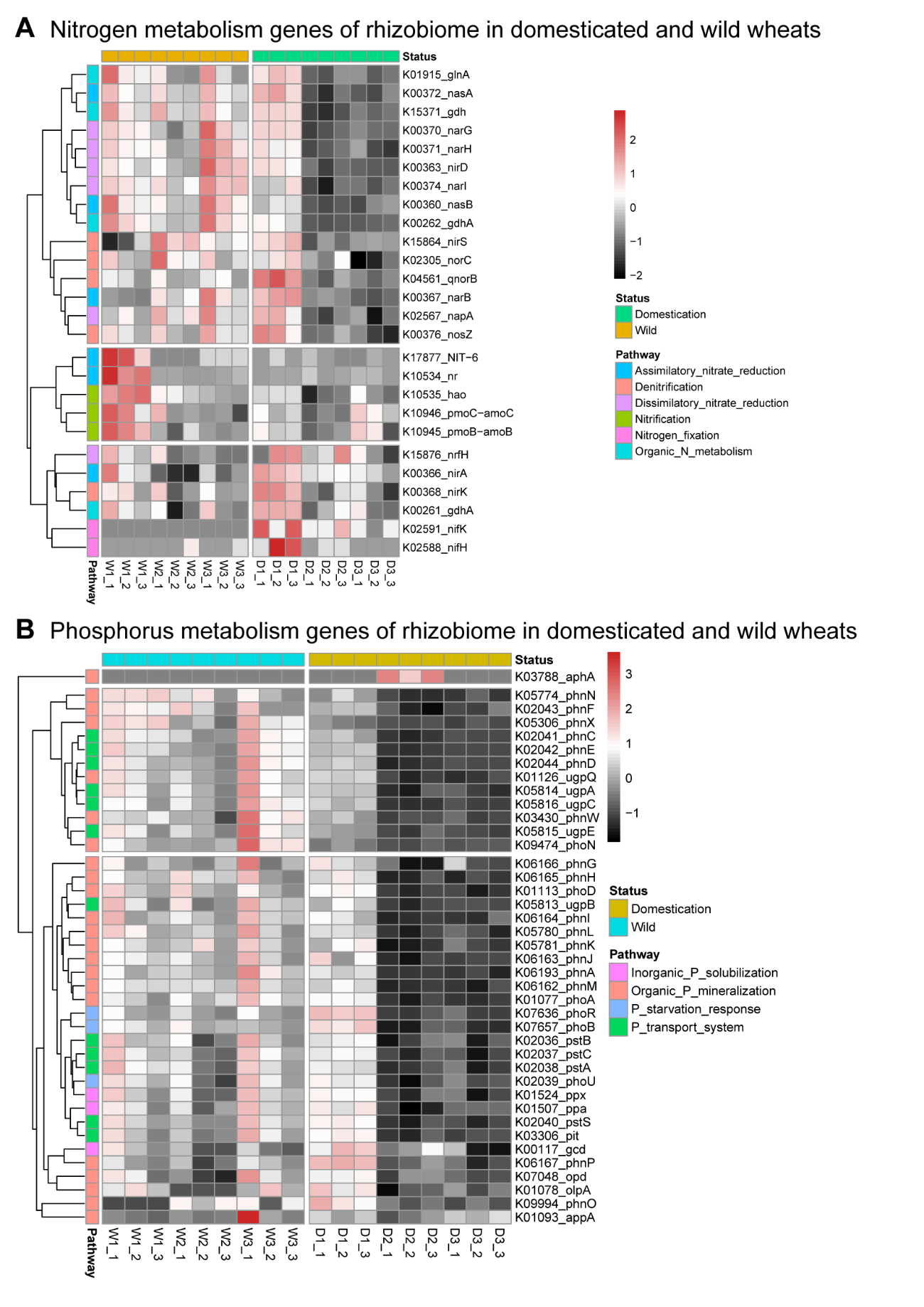
**

**Fig. S12** (A-H) Random forest (RF) mean predictor importance of KO functional categories as drivers for the plant phenotypes, including (A) plant height (PH), (B) chlorophyll content (CC), (C) ear length (EL), (D) subsegment length (SSL), (E) Tiller, (F) thousand grain weight (TGW), (G) seed length (SL), and (H) seed width (SW), respectively. The accuracy importance measure was calculated for each tree and averaged over the forest (2000 trees). Percentage increase in the mean squared error (MSE) of variables were applied to evaluate the importance of these predictors, and higher MSE% values represent more important predictors. Significance levels of each predictor are as follows: **P*< 0.05, ***P*< 0.01, and ****P*< 0.001.

**
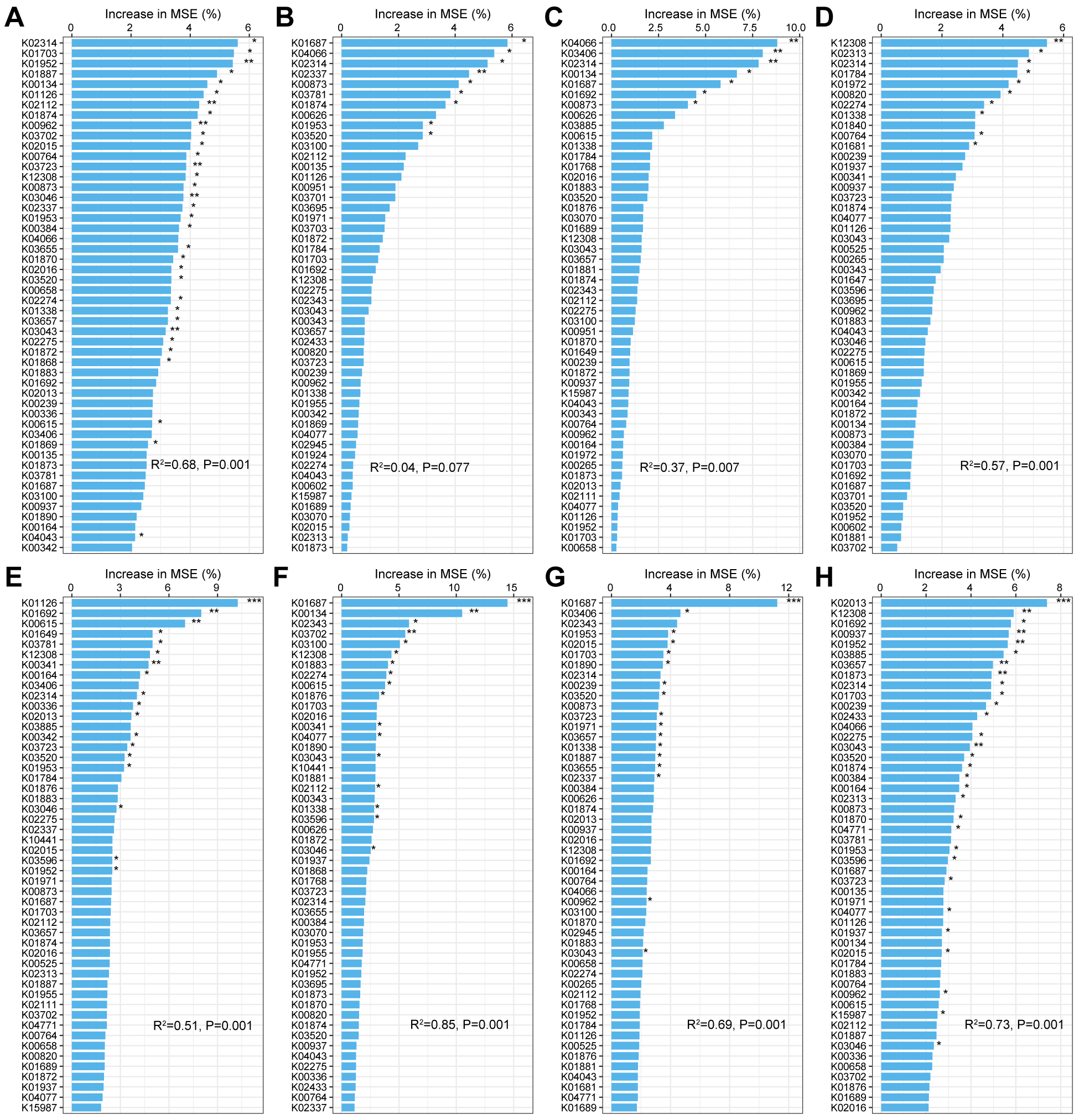
**

**Fig. S13** Partial least squares discriminant analysis (PLS-DA) and orthogonal partial least squares discriminant analysis (OPLS-DA) for root exudates that extracted from roots of wild and domesticated wheats.

**
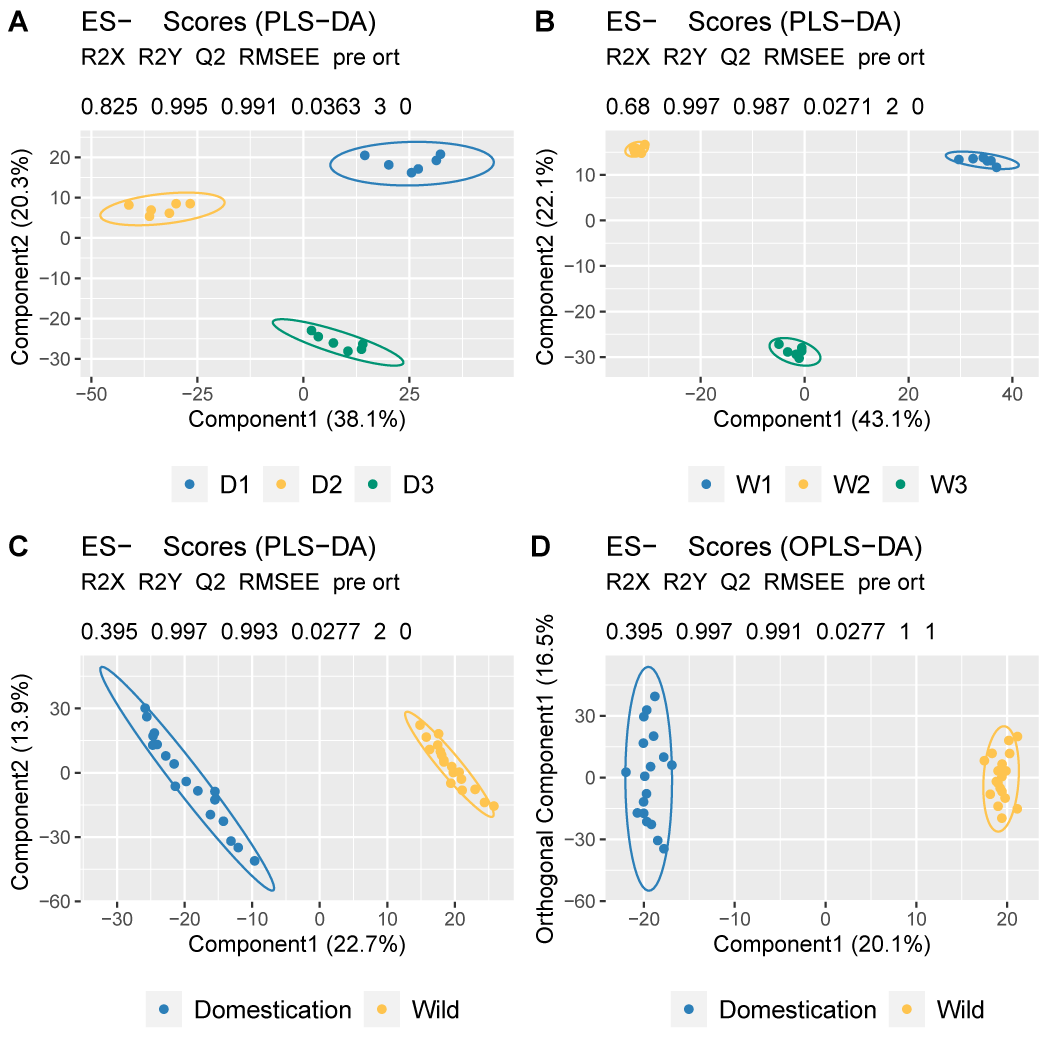
**

**Fig. S14** (A) The average relative abundance of enriched root exudates in the wild and domesticated wheats. (B) The fold changes of enriched metabolites in domesticated and wild wheats. The error bars represent standard errors of sample replicates and asterisks (*) indicate metabolic categories that are significantly more predominant in wild or domesticated wheats (*P* value <0.05, Wilcoxon test). (C) Heat map illustrating the relative abundance of root exudates in different accession of wheats.

**
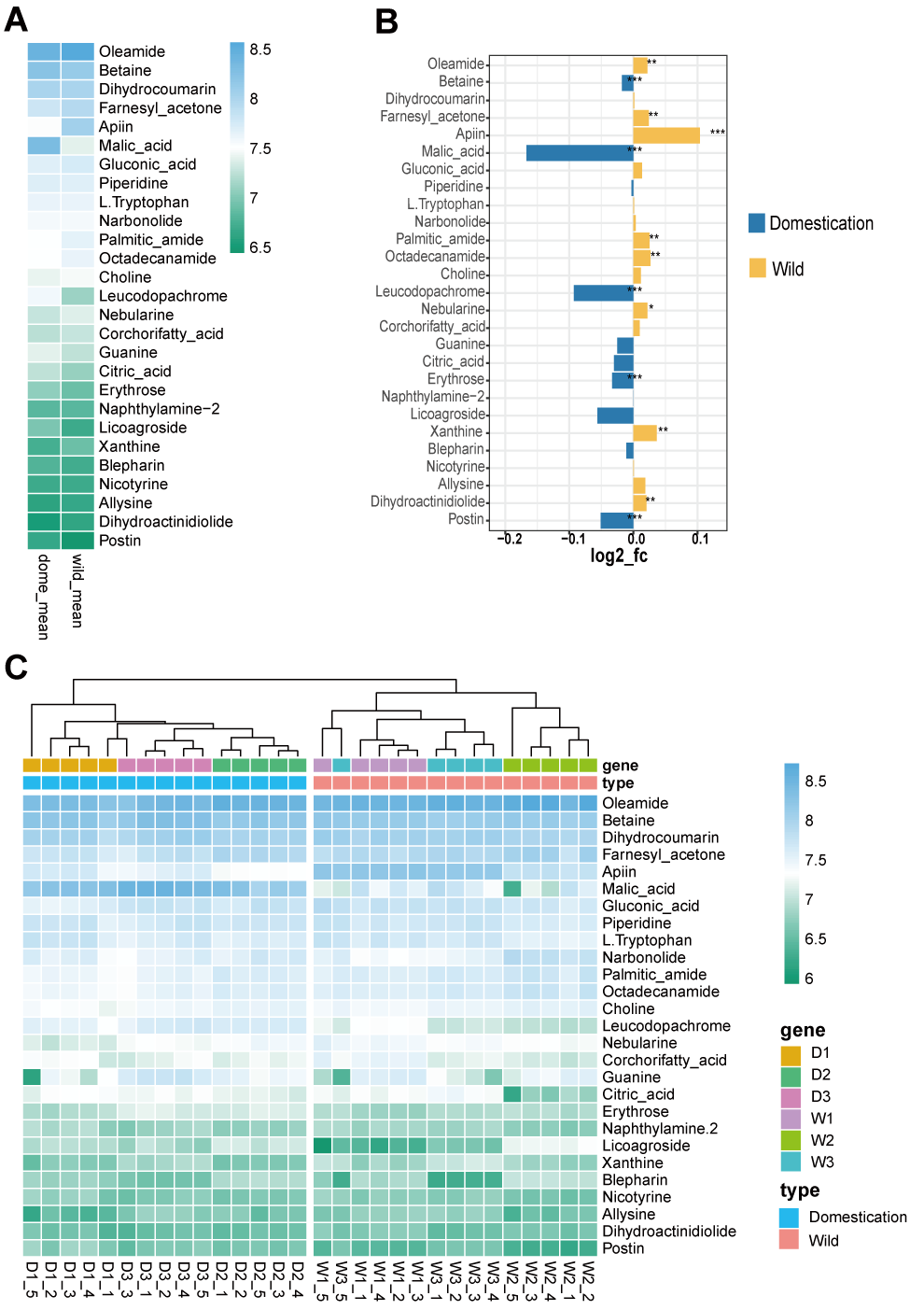
**

**Fig. S15** (A-H) Random forest (RF) mean predictor importance of enriched metabolites as drivers for the plant phenotypes, including (A) plant height (PH), (B) chlorophyll content (CC), (C) ear length (EL), (D) subsegment length (SSL), (E) Tiller, (F) thousand grain weight (TGW), (G) seed length (SL), and (H) seed width (SW), respectively. The accuracy importance measure was calculated for each tree and averaged over the forest (2000 trees). Percentage increase in the mean squared error (MSE) of variables were applied to evaluate the importance of these predictors, and higher MSE% values represent more important predictors. Significance levels of each predictor are as follows: **P*< 0.05, ***P*< 0.01, and ****P*< 0.001.


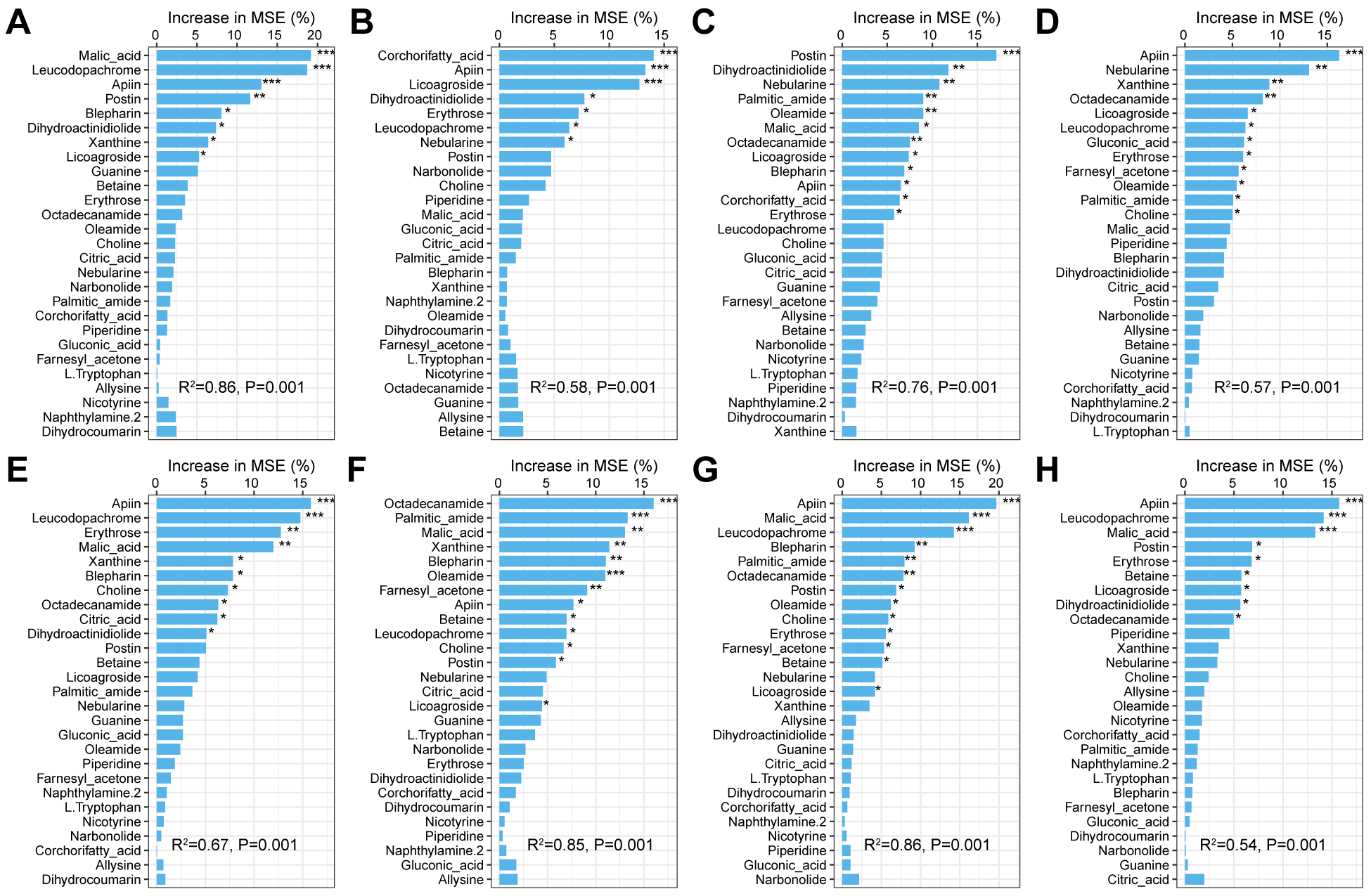


**Fig. S16** (A-D) Ecological associations between root morphology and rhizosphere bacteria and fungi in domestication wheat and wild wheats. Root morphology traits including root volume (RV), root average diameter (RAD), stem thickness (ST), root length (RL), fresh weight of root (FWR), were scanned and registered using Microtek ScanMaker i800 plus system. Significance levels of each association are as follows: **P*< 0.05, ***P*< 0.01, and ****P*< 0.001.


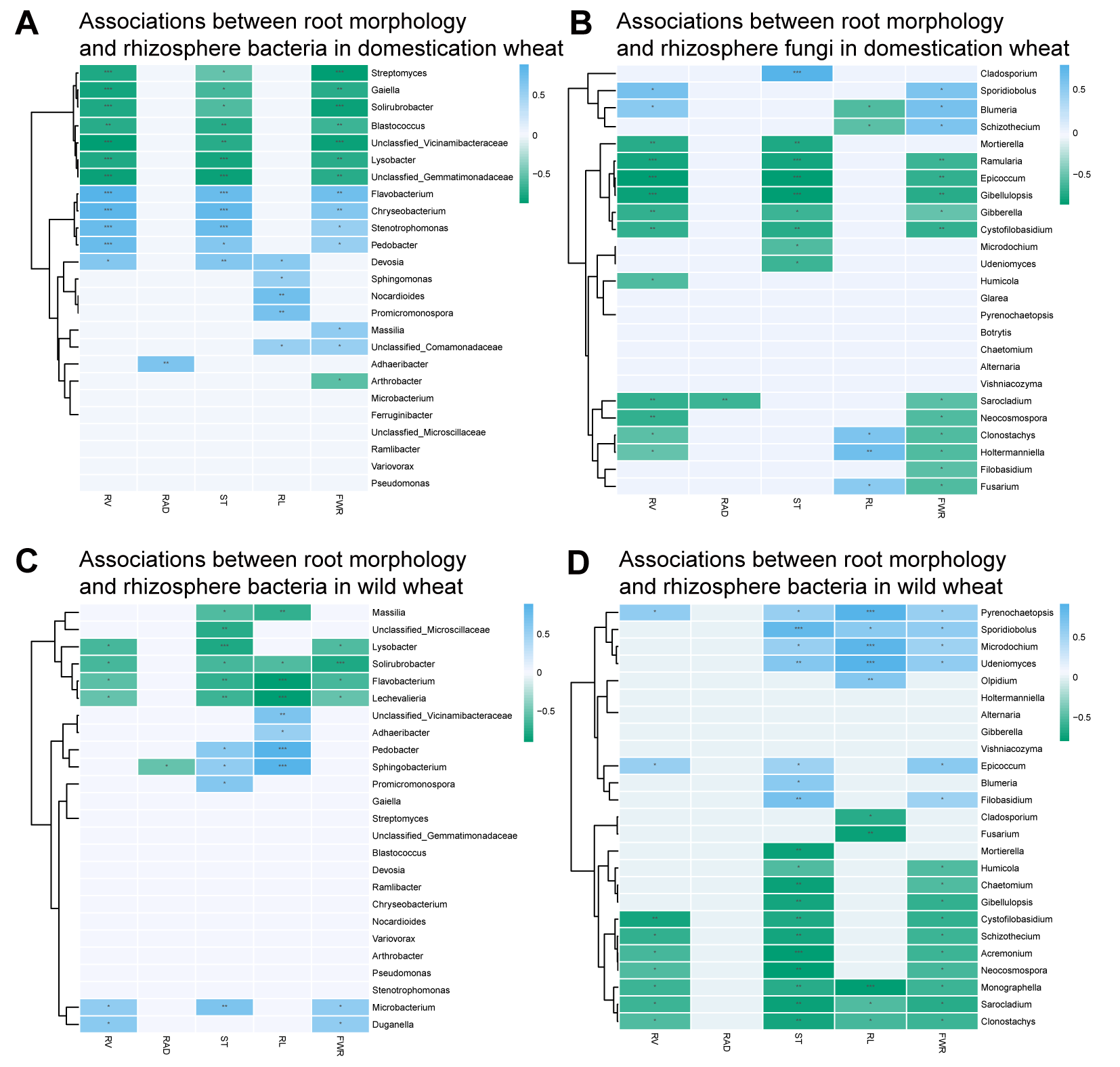


**Fig. S17** (A) Pearson correlation relationships between edaphic variables and carbon-cycling-related functional gene. (B) Pearson correlation relationships between dominant metabolites and carbon-cycling-related functional gene. Significance levels of each association are as follows: **P*< 0.05, ***P*< 0.01, and ****P*< 0.001.


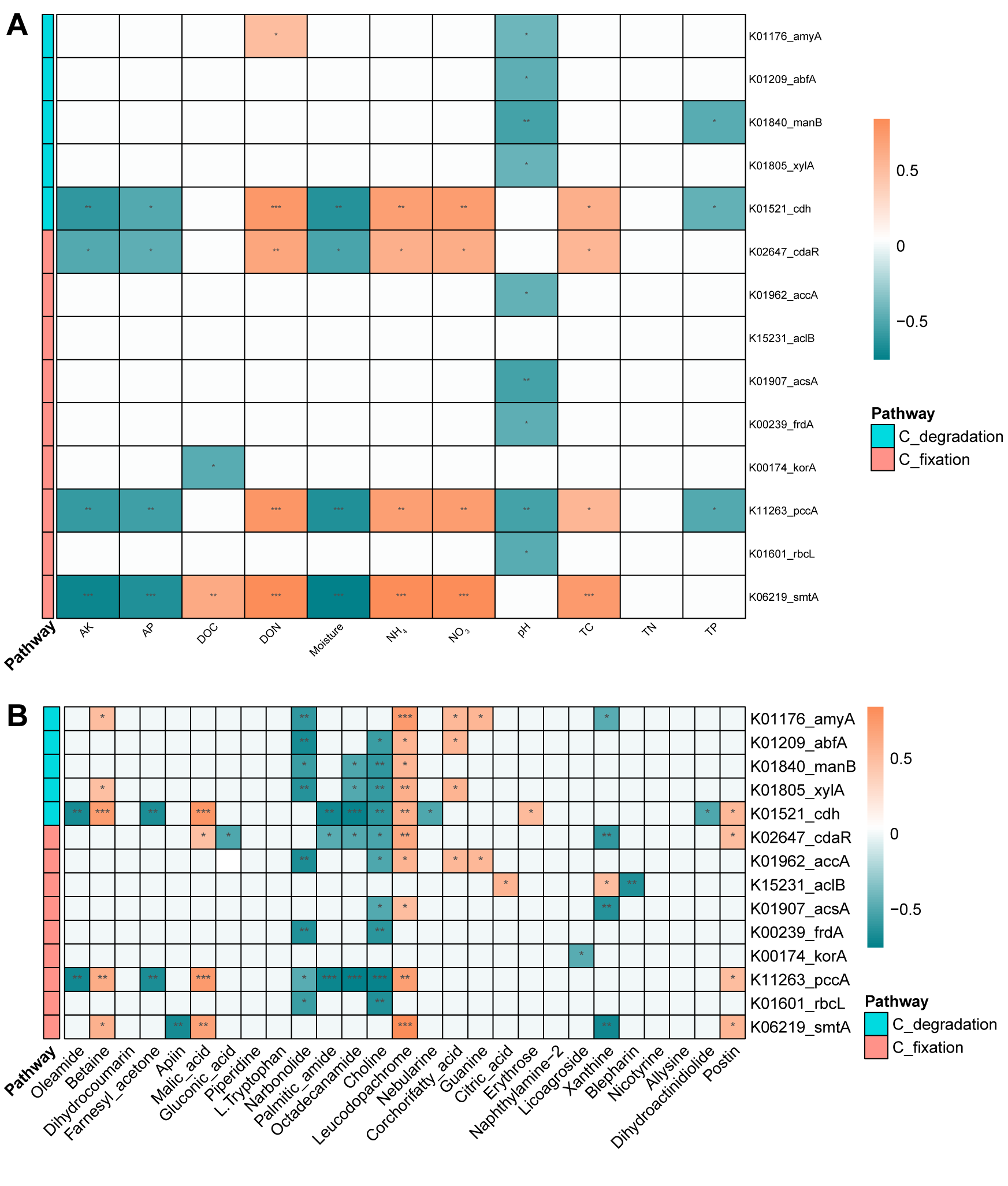


**Fig. S18** Pearson correlation relationships between dominant metabolites and phosphorus-cycling-related functional gene. Significance levels of each association are as follows: **P*< 0.05, ***P*< 0.01, and ****P*< 0.001.


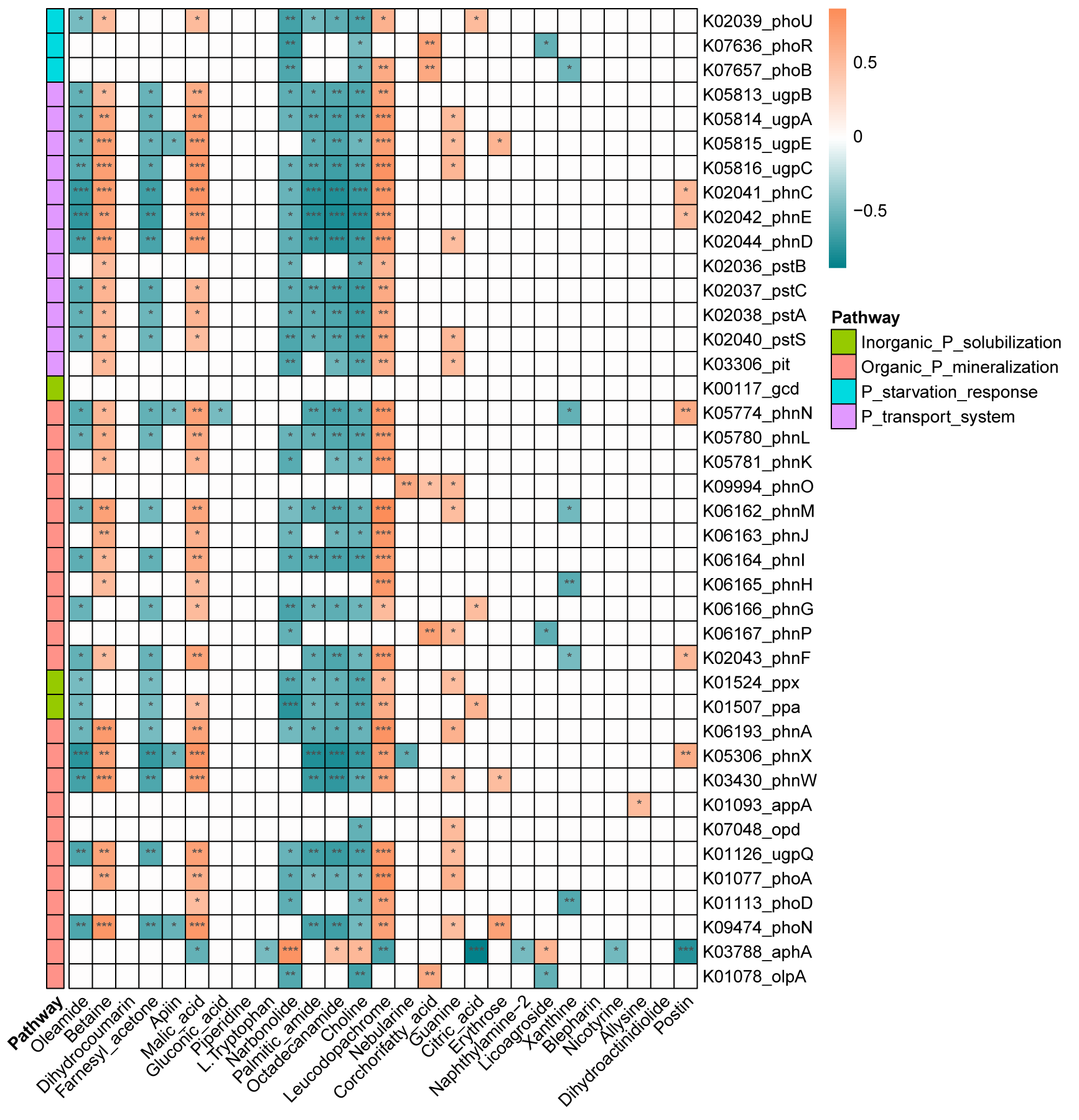


**Fig. S19** Pearson correlation relationship between dominant metabolites and nitrogen-cycling-related functional gene. Significance levels of each association are as follows: **P*< 0.05, ***P*< 0.01, and ****P*< 0.001.


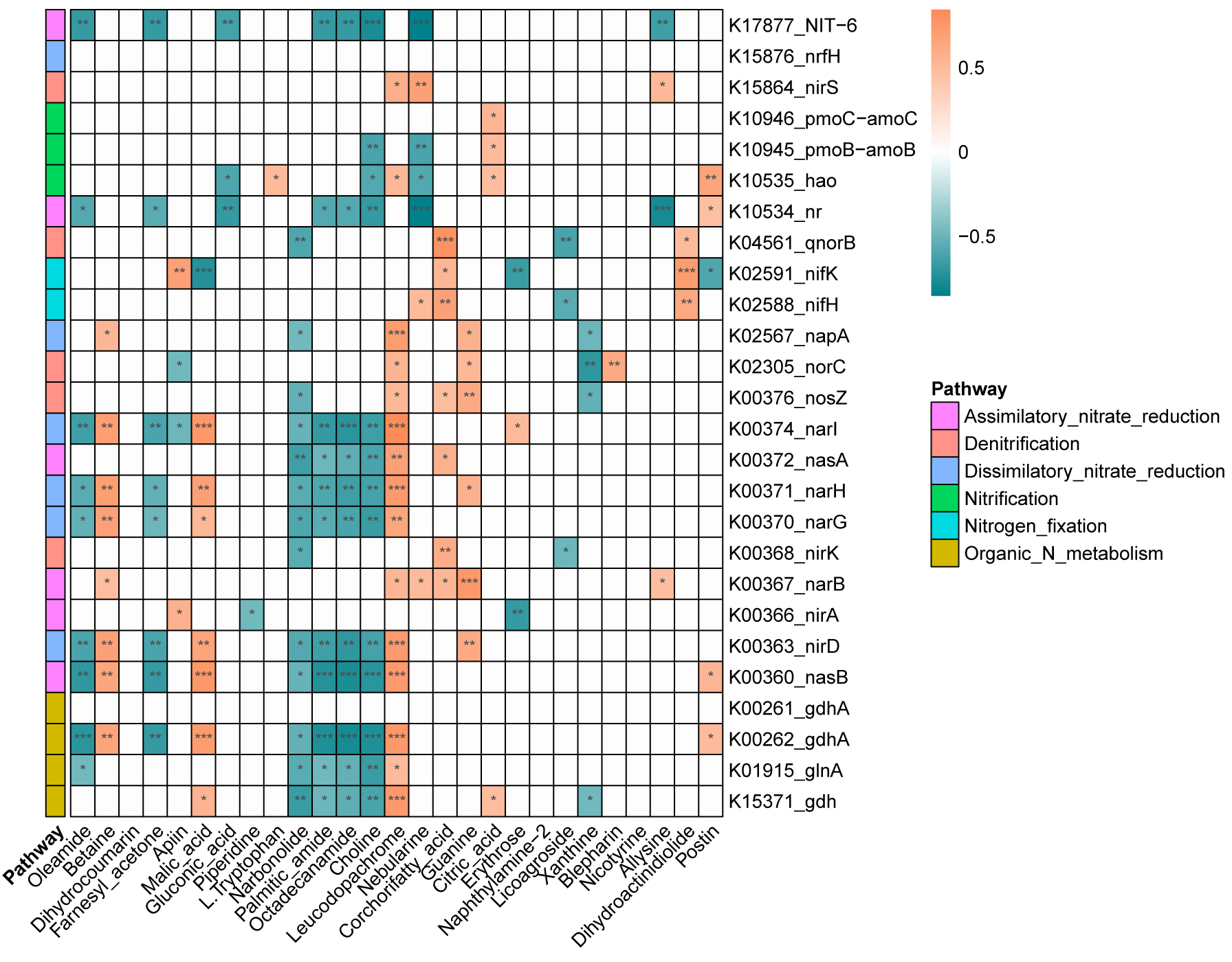

Supplement: Supplementary file 2 — Additional file 1: Fig. S1. Alpha diversity included Shannon index, Simpson index, ACE index, and Chao index for bacteria and fungi communities in different accession of domesticated and wild wheats. Errors bars represent standard errors (n= 5). Different lowcase letters above the bars indicate significant differences (P< 0.05), based on Kruskal-Wallis test. Fig. S2. The Bray-Curtis dissimilarity of bacteria and fungi communities in domesticated and wild wheats. DB1~DB3 and WB1-WB3 indicates that samples are affiliated with bulk soil in the domesticated and wild wheats, respectively. DT1~DT3 and WT1-WT3 indicates that samples are affiliated with rhizosphere soil in the domesticated and wild wheats, respectively. The dots represent the values of Bray-Curtis dissimilarity between replicates in the any of groups. The details on the D1-D3 and W1-W3 groups are available in Table S2. Fig. S3. Constrained Analysis of Principal Coordinates analysis (CAP) ordination constraned to domestication status (left panel), habitat type (middle panel), and genome group (right panel) based on Bray-Curtis metric of bacteria and fungi communities. Variance of community dissimilarity among five treatment were draw from ANOVA-like permutation analysis. Fig. S4. Principal coordinate analysis ordinations (PCoA) for bacteria and fungi communities in domesticated and wild wheats. Fig. S5. Distribution of dominant phyla and genus in the bacteria and fungi communities in domesticated and wild wheats. Fig. S6. Amplicon sequences variants (ASVs) form bulk soils responsible for the community differences in the wild wheats and domesticated wheats that are calculated by a differential abundance test and random forest classification. (A) and (B) The volcano plot illustrating the enrichment and depletion patterns of rhizosphere bacterial and fungal microbiomes in the three wild wheats compared with three domesticated wheat accessions. DI, depleted index; DSI, dissimilarity index. (C) and (D) Joyplots s [file 40168_2023_1513_MOESM1_ESM.docx]
