# Supplementary material for: Plant domestication shapes rhizosphere microbiome assembly and metabolic functions
Source: Microbiome. 2023 Mar 31;11:70. doi: 10.1186/s40168-023-01513-1 (PMC10064753; doi:10.1186/s40168-023-01513-1)
Supplement: Supplementary file 4 — Additional file 3: Method S1. Experiment design and field site management. Method S2. Samples collection, DNA extraction, PCR amplification and amplicon sequencing. Method S3. Shotgun metagenomics sequencing. Method S4. Root exudate collection and LC-MS analysis. Method S5. Microbiota inoculation experiment. Method S6. Statistical analyses. [file 40168_2023_1513_MOESM3_ESM.docx]

**Supplementary Notes**

**Plant domestication shapes rhizosphere microbiome assembly and metabolic functions**

Hong Yue^1#^, Wenjie Yue^1#^, Shuo Jiao^2,3^, Hyun Kim^4^, Yong-Hwan Lee^4^,

Gehong Wei^2,3 ***^, Weining Song^1 **^, Duntao Shu^2,3 *^

^1^ College of Agronomy, State Key Laboratory of Crop Stress Biology in Arid Areas, Northwest A&F University, Yangling, Shaanxi 712100, China

^2^ Shaanxi Key Laboratory of Agricultural and Environmental Microbiology, Yangling, Shaanxi 712100, China

^3^ State Key Laboratory of Crop Stress Biology in Arid Areas, College of Life Sciences, Northwest A&F University, Yangling, Shaanxi 712100, China

^4^ Department of Agricultural Biotechnology, Seoul National University, Seoul 08826, Korea

^#^ These authors contributed equally to this work

^*^ Corresponding author: [donald.shu@nwafu.edu.cn](mailto:donald.shu@nwafu.edu.cn) (D. Shu)

^**^ Corresponding author: sweining2002@yahoo.com (W. Song)

^***^ Corresponding author: [weigehong@nwafu.edu.cn](mailto:weigehong@nwafu.edu.cn) (G. Wei)

Tel: 86-18710994335; Fax: 86-29-87090162

**1. Extended Methods**

**Method S1** Experiment design and field site management.

**Method S2** Samples collection, DNA extraction, PCR amplification and amplicon sequencing

**Method S3** Shotgun metagenomics sequencing

**Method S4** Root exudate collection and LC-MS analysis

**Method S5** Microbiota inoculation experiment

**Method S6** Statistical analyses

**1. Extended Methods**

**Method S1** Experiment design and field site management.

To explore the initial evolutionary history of these tetraploid wheat varieties, a phylogenetic tree was constructed based on their whole-genome sequences. The MEGA-X program (https://www.megasoftware.net/) was applied to generate a neighbor-joining tree with 1000 bootstraps.

The field experiment for this study was managed with the rigorous and consistent winter-wheat summer fallow system in the last nine years, as well as fertilization regimes comprising urea (60 kg N ha^-1^), superphosphate (100 kg P_2_O_5_ ha^-1^ year^-1^), and potassium chloride (90 kg KCl ha^-1^ year^-1^). The domestication experiment for 44 tetraploid wheat accessions was established since 2014 at Caoxingzhuang Agro-Ecosystem Experimental Station of the Northwest Agriculture and Forest University, Shaanxi Province, China (34°17′N, 108°04′E). The site has an elevation of 511 m and the typical soil is Huangmian (Calcaric Cambisols, FAO). The annual precipitation in this region is 635.1 mm with the annual mean temperature of 12.9°C.

The field layout with completely randomized design included plant genotypes and replications. In this study, there are 528 plots in total for all 44 tetraploid wheat accessions. Each plant genotypes have five replicate plots. Each plot in this filed experiment is 3 m^2^ with 2 m width and 1.5 m length. The width of 1 m space were set as guard rows between plots. For irrigation scheme, multiple irrigation strategies were used in different plant development stage, according to the “Quotas for agricultural irrigation water: wheat” that was released by our government in 2014. Briefly, from the germination stage to seedling stage in the early of October to later of October, no irrigation scheme was arranged. At the tillering stage from the early of November to the middle of December, the irrigation water capacity is usually ~450-600 m^3^/ha for one day. Then, no irrigation schemes were arranged during the over-winter period. At the jointing stage (the middle of March to the middle of April), the irrigation water capacity is usually ~750-900 m^3^/ha for one day. From the heading period (the latter of April) to grain filling period (the latter of March), the irrigation water capacity is usually ~450-600 m^3^/ha for one day. Taken together, at the latter of wheat development stage, the irrigation scheme (~450-600 m^3^/ha for one day) will be arranged when the maximum filed capacity is less than 60%.

It should be noted that the goals of domestication of wheat and plant breeding is to select high-yield accessions for promoting food provision. We could not got enough crop yield to meet an increasing demand of food for our humans if the wheat without herbicide and pesticide addition that it is widely acknowledged by the worldwide scientific researchers and farmers. In order to avoid the competition for soil nutrients from weeds, herbicide was used to preventing and removing annual in the wheat field. After the stage of 4 leaf sheaths lengthen, ~1.87-3.75 kg/ha of isoproturon (50%) or 900-1200 mL of pinoxaden (5%) was nebulized to the wheat filed. Considering the occurring of plant disease occur during the whole plant development stage could lead to the huge yield loss, some pesticide has been successfully applied to protect the wheat from the disease, such as shape eyespot and wheat yellow rust. Cypermethrin and triazole fungicide were widely used in the wheat field in the Caoxingzhuang Agro-Ecosystem Experimental Station.

**Method S2 DNA extraction, PCR amplification and amplicon sequencing**

For samples collection, plants in each wheat accession group were randomly selected and carefully removed from field locations with intact roots. The loosely root-attached soil was removed by vigorous shaking, and subsequently, the entire root system was transferred to a 15 ml tube containing 5 ml of Life Guard Soil Preservation Solution (Mo Bio Laboratories, Carlsbad, CA, USA). The tubes were vigorously shaken and then centrifuged at 14,000×g for 1 minute to collected ~1g of rhizospheric soil per replicate for genomic DNA extraction. For bulk soils, topsoils from the inter-row without plants (at 10~20 cm depth) were collected and homogenized, and then approximately 1 g bulk soils were also submerged in LifeGuard solution. In total, 60 soil samples were collected (5 replicates × 6 plant accessions × 2 compartments). All soil samples were stored at -30°C until further use.

For DNA extraction, three independent DNA extractions of each soil sample were pooled to generate one homogenized single DNA sample to minimize any DNA isolation bias. The concentration of extracted DNA was measured by a Nanodrop Spectrophotometer ND-2000 (Thermo Fisher Scientific, USA), and the quality was further checked by 1.0% agarose gel electrophoresis.

For PCR amplification, the universal 515F (5’-GTGCCAGCMGCCGCGGTAA-3’) and 806R (5’-GGACTACHVGGGTWTCTAAT-3’) PCR primers with barcodes were applied to amplify the V4 regions of 16S rRNA. The PCR primers ITS1F (5’-CTTGGTCATTTAGAGGAAGTAA -3’) and ITS2R (5’-GCTGCGTTCTTCATC-GATGC -3’). After sequencing, the DADA2 plugin in QIIME2 was then used to denoise and generate an amplicon sequence variants (ASVs) count table for downstream bioinformatics analysis. The taxonomic assignment was performed using the q2-feature-classifier in QIIEM2 via the Silva SSU database (Release 138, <http://www.arb-silva.de>) for bacteria and the UNITE database for fungi at a 90% bootstrap confidence level.

**Method S3 Shotgun metagenomics sequencing**

For taxonomic assignment, the non-redundant genes were then searched against the NCBI NR database (release March 15, 2020) using DIAMOND (v0.922.123) with an E value cutoff of 1e^-5^ ([Buchfink et al., 2015](#_ENREF_5)). Reference protein IDs of best hits were deployed to disentangle the taxonomic affiliation. For functional annotation, the numbers of reads mapping to genes for each sample were calculated using Bowtie2 ([Langmead & Salzberg, 2012](#_ENREF_17)). Then, the relative abundance of non-redundant genes in each sample, which was expressed as transcripts per kilobase million (TPM), was further measured based upon the mapped reads and length of genes.

**Method S4 Root exudate collection and LC-MS analysis**

For root exudate collection, root samples were further washed thoroughly with Milli-Q water to remove all adhering soil. Simultaneously, dead and broken roots were trimmed using stainless steel tweezers. Each group of plants (four to six plants) was then transferred to a 200 ml soil solution that was generated from a 1:10, soil: Milli-Q water mixture and arranged in a growth chamber for 48 h in darkness. After 48 h, each plant group was rinsed and transferred to a conical flask with 200 ml sterilized deionized water for 2 h at 120 rpm at ambient temperature and in the dark to collect root exudates. The supernatants in the flask were subsequently filtered through a 0.22 μm membrane filter, and 45 ml of each root exudate solution was further freeze-dried for 72 h in a freeze dryer (Christ Alpha2-4 LSC, Germany) to obtain root exudate powders. Subsequently, these powders were carefully transferred to 15 ml vials for chromatography-mass spectrometry (LC-MS) analysis.

An ExionLCTMAD system (AB Sciex, USA) equipped with an ACQUITY UPLC BEH C18 column (100 mm× 2.1 mm; 1.7 µm; Waters, Milford, USA) was used to perform chromatographic separation of metabolites. The mobile phases comprised 0.1% formic acid in water and 0.1% formic acid in an acetonitrile: isopropanol mixture (1:1, v/v). The 10 μL of root exudate solution was eluted with mobile phases at a flow rate of 0.4 mL/min at 40°C, and positive (ESI+) and negative (ESI-) ion modes in the UPLC system were used to detect metabolites. Progenesis QI (v2.3, Nonlinear Dynamics, Waters, USA) was used to obtain peak intensity, retention time (RT), and mass-to-charge ratio (m/z) values.

**Method S5 Microbiota inoculation experiment**

In the current study, we conducted a two-level factorial experiment (inoculation treatment × plant variety) to investigate whether rhizosphere microbes (control vs. inoculation) can influence the morphologic traits of domesticated and wild wheats. For this experiment, each live microbiota suspensions being specific to each genotype. For instance, the live microbiota suspensions from wild variety #1 (W1) rhizosphere soil were inoculation to wild variety #1 (W1), while the live microbiota suspensions from domesticated variety #1 (D1) rhizosphere soil were inoculation to domesticated variety #1 (D2). The common method used to estimate bacterial load is reading optical density (OD) at 600 nm. The OD method can be performed automatically in a high throughput manner using a microtiter plate reader. Consistent with previous study, the inoculation treatments were added 2.0 ml live microbiota suspensions with 0.5 value of OD600, which corresponds to ~8×10^8^ CFU/mL of cell count. As for the selection of *Microbacterium* *mitrae*, we firstly found that the genera *Microbacterium* present contrasting associations with plant phenotypes and enriched metabolites between domesticated and wild wheats.

Based on this results, it was safely postulated that the genera *Microbacterium* not only played a key role in sustaining plant growth under field conditions but also had potential contribution to root morphological traits under experimental conditions. Furthermore, on the base of results from the MWAS, isolation rhizosphere-derived bacteria were conducted. After isolation, we obtained 22 bacteria strains. By taking the intersection between isolated bacteria strains and putative keystone taxa from statistical analyses, seven bacteria strains included *Microbacterium mitrae* were finally obtained to conduct single microbiota inoculation experiment. We therefore select *Microbacterium mitrae* to conduct downstream experiment. As expected, *Microbacterium mitrae* showed most strongly observations in the aspect of root morphology traits, including seeding fresh weight, dry weight, root length, and root average diameter. For the selection of culture medium, soil substrate is usually used in the research of Arabidopsis due to their short length, as reported elsewhere ([Lv et al., 2022](#_ENREF_19)). The Hoagland solution was one of the most popular nutrient solutions, which were widely used to explore the root growing of wheat. Considering the root length is more than 20 cm, compared to soild substrate, Hoagland’s nutrient solution showed more convenience and was therefore select to observe the root morphology traits. The cross inoculation, which means transplant the rhizosphere consortia from wild to domesticated, does not easily to distinguish the contribution of multiple plant genotypes or single inoculum to root growth in the current study. Furthermore, given the existence of *Microbacterium mitrae* in both the rhizosphere of domesticated and wild wheats, the self inoculation was therefore applied to explore the role of *M. mitrae* in maintaining root growth of wheats.

For rhizospheric microbiota inoculation, live microbiota suspensions were generated by mixing 5 g of rhizospheric soil into 50 mL TSB medium, and shaken at 60 rpm for 2 d at 28°C. After that, the supernatants were collected and further shaken at 60 rpm for 3 d. The inoculation treatments were added 2.0 ml live microbiota suspensions with 0.5 value of OD600, and without addition of rhizospheric microbiota were denoted as control treatment. *M. mitrae* was grown on R2A medium and incubated at 28°C with shaking at 250 rpm until bacteria culture to OD600 = 1. This bacterial culture was centrifuged at 4,000 g for 10 min and washed twice with 10 mM MgCl_2_, and resuspended in full-strength of Hoagland’s nutrient solution (Phygene Biotechnology Co. Ltd, CN).

**Method S6 Statistical analyses**

In this study, all statistical analyses were conducted using R software (v 4.0.1) ([Team, 2013](#_ENREF_29)). Significant differences among groups were calculated by Kruskal Wallis tests using the “agricolae” R package ([de Mendiburu, 2019](#_ENREF_7)). P values were corrected by using the “fdrtool” R package ([Strimmer, 2008](#_ENREF_27)) and P_adjust_ (q) < 0.05 represented significance. For a-diversity, four estimators included Shannon index, inverse Simpson index, ACE richness, and Chao1 richness, were calculated based on the subsampling of the ASV feature table. For beta-diversity, an analysis of similarity (ANOSIM) was implemented by the “anoism” function to elucidate the pairwise similarity between groups in bacterial and fungal communities. Principal coordinate analysis (PCoA) was performed to elucidate community dissimilarity between groups by using the “capscale” function. Permutational multivariate analysis of variance (PERMANOVA) retrieved from the “adonis” function was used to measure the effect of variables and significance on β-diversity. To determine the enriched and depleted ASVs in wild and domesticated wheat plants, differential abundance analysis was conducted using the generalized linear model (GLM) approach in the “EdgeR” package. Additionally, the depleted (DI) and dissimilarity index (DSI) were calculated to assess microbial selection processes from wild to domestication and from bulk soil to rhizosphere soil.

For Random forest classification, two-thirds of the total samples in the whole dataset were randomly selected as the training set, and the remaining samples were defined as testing set in the RF model. ASVs were arranged according to their importance in contributing to the classification accuracy of domesticated status prediction in the RF model by calculating the mean decrease accuracy.

For microbial co-occurrence network analysis, to explore the microbial hierarchical interactions between bacterial and fungal communities in the rhizosphere of wild and domesticated wheats, we implemented correlation-based network analysis. The FastSpar algorithm ([Watts et al., 2019](#_ENREF_32)), an efficient and scalable correlation estimation of the SparCC for compositional datasets, was used to construct a microbial co-occurrence network with 50 iterations and 1000 bootstrap correlations. To minimize the bias of pairwise correlations, the core community for bacteria was generated based on the criteria of mean relative abundance (MRA) > 0.04% and occurrence frequency > 80% across all samples. For fungi, the core community was constructed upon MRA >2% and occurrence frequency >60% in all samples. Meanwhile, 1000 Erdös–Réyni random networks with the same number of nodes and edges as the real network were also generated using the “igraph” package. For random and empirical networks, global topology features such as the modularity value, average path length, and average clustering coefficient were also measured. Network modules were characterized using the “cluster_fast_greedy” function in the “igraph” package. For the definition of keystone taxa in the rhizosphere network of wild and domesticated wheat, ASVs with degrees greater than 8, node transitivity greater than 0.5, and betweenness centrality lower than 2000 were defined as keystone ASVs. For the bulk soil network of wild and domesticated wheat, ASVs with a degree greater than 10, node transitivity greater than 0.5, and betweenness centrality lower than 1000 were defined as keystone ASVs.

Finally, we used plant-microbiota-metabolite association analysis to elucidate the difference in metabolite features between wild and domesticated wheat, the relative abundance of plant-derived metabolites > 6 was selected and clustered along the domestication status and plant genotype gradient. The metabolites with variable importance in projection (VIP) ≥ 1 and q value (Wilcoxon rank-sum test) < 0.05 were further selected as significant metabolites for downstream analysis. Based on this association analysis, key relevant metabolites that were significantly enriched in wild or domesticated wheat were used to evaluate their importance for plant growth. These key metabolites were used as independent variables, whereas each plant trait was used as a dependent variable.
